# Popularizing health education, building public health facilities or regular screening? How to prevent schistosomiasis more effectively in African children

**DOI:** 10.1371/journal.pone.0347325

**Published:** 2026-04-20

**Authors:** Shansong Wu, Yuntao Bai, Jiahao Li, Yueling Yang

**Affiliations:** 1 School of Accounting, Wuxi Taihu University, Wuxi, China; 2 Business School, Shandong Management University, Jinan, China; 3 Information Engineering School, Shandong Management University, Jinan, China; 4 Department of Economics and Rural Development, Gembloux Agro-Bio Tech, University of Liège, Gembloux, Belgium; University of Uyo, NIGERIA

## Abstract

Due to the lack of safe drinking water and public health facilities, as well as the low popularity of health education, schistosomiasis is a prominent problem in African children. Considering that the process of schistosomiasis prevention in African children is dynamic and continuous, this paper uses a differential game model to study how to prevent schistosomiasis in African children. Common prevention modes of schistosomiasis in African children include universal health education, construction of public health facilities, and regular screening. This paper constructs a differential game model under these three modes, and compares and analyzes the equilibrium results. Finally, the study concludes that when the benefits obtained by government and social forces from preventing schistosomiasis at a unit level are small, universal health education mode can make the government and social forces obtain the maximum social benefits, followed by the establishment of public health facilities mode, and the minimum social benefits obtained by regular screening mode. With the increase of the benefits obtained by government and social forces from preventing schistosomiasis at a unit level, the social benefits obtained by government and social forces from regular screening mode decrease first and then increase. When the benefits obtained from preventing schistosomiasis at a unit level increase to a certain extent, the social benefits obtained by regular screening are the maximum.

## 1. Introduction

Schistosomiasis is a serious public health problem in Africa especially among children in some of the poorer parts of the continent. Schistosomiasis is a parasitic disease caused by Schistosoma haematobium. According to the World Health Organization (WHO) Africa Report, in 2023, the total number of people requiring preventive chemotherapy globally is 253.8 million, 135 million of whom are school-age children. Data on treatment of school-aged children in 33 countries and treatment of adults in 20 countries reported in 2023 show that approximately 90 million people worldwide received prophylactic chemotherapy for schistosomiasis, which equates to 56% of global coverage of school-aged children [1]. Schistosomiasis is a serious public health problem in Africa especially among children in some of the poorer parts of the continent. It is is the second most important tropical disease in the world [2]. Schistosomiasis is a parasitic disease caused by Schistosoma haematobium. People are exposed to the pathogen through contact with fresh water contaminated with Schistosoma larvae, such as: swimming, fishing, bathing, or other water activities [3]. The pathogen of schistosomiasis – schistosoma, mainly causes severe damage to these organs by parasitizing in the human intestinal or urinary system. In children, the disease may cause a range of health problems, including anemia, malnutrition, liver disease, and chronic illnesses that may lead to organ damage. Studies have found that schistosomiasis spinalis may also adversely affect children’s height, skull size and cognitive abilities.

There are many reasons for the high incidence of schistosomiasis among African children. First, there is a lack of access to safe drinking water and basic sanitation [4]. Second, the frequency of exposure to contaminated water sources is high. Thirdly, there is limited awareness of schistosomiasis and a lack of effective preventive and early diagnostic measures [5]. Fourth, due to economic constraints, medical resources are very limited, and effective prevention and treatment measures are often difficult to implement [6]. This makes it difficult for children to receive timely and effective treatment once they are infected. Fifth, the particular ecological environment of some regions, such as the tropical climate, favors the survival and reproduction of schistosomes, raising the risk of infection [7]. Therefore, to reduce the incidence of schistosomiasis in children, it is necessary to take a comprehensive series of public health measures, such as the provision of safe drinking water and washing water sources, strengthening public health education, raising people’s awareness of disease prevention and control, and improving medical conditions.

In order to more effectively prevent schistosomiasis in African children, a number of measures can be taken. First, ensure clean and safe drinking and domestic water and reduce people’s exposure to water sources contaminated by schistosome larvae [8]. Second, strengthen health education and publicity on schistosomiasis, raise children’s and parents’ awareness of schistosomiasis and its prevention and treatment, and learn to avoid high-risk activities. Third, improve water resource management, upgrade basic sanitation facilities, such as building sanitary latrines, and reduce the direct discharge of sewage to reduce the risk of schistosomiasis transmission [9]. Fourth, screen children for schistosomiasis annually or at regular intervals, and provide timely drug treatment to those infected [10]. The use of anti-schistosomal drugs such as Praziquantel can be promoted. Fifth, establish an effective outbreak monitoring and reporting system to help health authorities keep abreast of outbreak developments and focus attention on high-risk areas. Sixth, encourage community residents to participate in schistosomiasis control activities and increase the control capacity of communities and households [11]. By implementing these measures, it is possible to create a healthier living environment for African children and reduce the incidence and impact of schistosomiasis.

Some scholars have studied the causes of schistosomiasis transmission. For example, Ping et al. (2014) studied the transnational transmission of schistosomiasis in China [12]. Rubaba et al. (2016) concluded that snail predation is an important cause of schistosomiasis transmission [13]. These causes include both transnational and food-related factors.

Some scholars have studied the harm caused by schistosomiasis. For example, schistosomiasis causes an increase in the number of lung nodules [14]. Schistosomiasis can lead to infection of the central nervous system in children [15]. These studies include the effects of schistosomiasis on lung nodules and the central nervous system.

In the face of the dangers of schistosomiasis, some scholars have researched drugs for the treatment of schistosomiasis. For example, Stothard (2013) concluded that praziquantel is effective in treating schistosomiasis [16]; Roucher et al. (2021) conducted trials to validate artesunate-mefloquine as an alternative to schistosomiasis in African children [17]; and Lothe et al. (2018) argued that improved hygiene literacy reduces schistosomiasis incidence [18]; Stothard et al. (2011) believed that Praziquantel (PZQ) can help treat schistosomiasis in African children [19]. These studies cover the role played by different drugs in the management of schistosomiasis.

In managing schistosomiasis among African children, comprehensive management tools prove more effective than drug research alone, for several reasons. First, control requires not only effective drugs but also measures to interrupt transmission, such as clean water provision, improved sanitation, water resource management, and health education. Second, while drugs offer short-term solutions, management tools like water quality improvement and community education provide sustainable and holistic outcomes. Third, over-reliance on drugs risks resistance and often overlooks asymptomatic carriers, who continue to spread the disease. Fourth, drug development is costly and resource-intensive, whereas infrastructure investments are more cost-effective and benefit entire communities. Finally, management measures, such as clean water access and excreta management, achieve broader population coverage than treatment programs, which often target limited groups [4]. Thus, while drug treatment is essential, integrating public health and socio-economic management measures yields wider and longer-term impacts.

Some scholars have studied how to manage infectious diseases from a management perspective. For example, in terms of universal health education, health education in schools can help reduce the spread of infectious diseases [20]; and the impact of online program learning on the spread of new coronaviruses [21]. In the context of public health infrastructure development, some studies have investigated the role of public health infrastructure in cases of Acute Encephalitis Syndrome in India [22], as well as whether the expansion of public facilities could mitigate the transmission of NeoCoronavirus in remote mountainous regions of Alaska [23]. In terms of enhanced surveillance, multidimensional heterogeneous population screening is an important tool for detecting infectious diseases [24]. Active screening can screen for tuberculosis in refugees [25]. Pregnancy screening screens children exposed to hepatitis C virus [26]. Abe et al. (2020) argue that the Chinese experience should be drawn upon and that effective monitoring and surveillance system mechanisms will facilitate effective and locally-tailored management of schistosomiasis elimination [27]. The research of these scholars on the prevention of other infectious diseases can inform the prevention of schistosomiasis in African children.

In recent years, schistosomiasis, as a public health issue in Africa, has garnered widespread attention regarding its transmission mechanisms and control strategies. African scholars have explored the key factors influencing the spread of schistosomiasis from various perspectives, providing a crucial foundation for formulating effective prevention and control measures. Sule et al. (2025) concluded through analysis that an integrated and coordinated approach is essential for successfully controlling the transmission risks of schistosomiasis associated with water resource development [28]. Asare et al. (2025) investigated the impact of climate change on the transmission of schistosomiasis [29]. Reigl et al. (2025) analyzed the socio-cultural and structural barriers affecting parents’ understanding and access to information about schistosomiasis among children around Ugandan lakes [30]. Mwinzi et al. (2025) examined the priority knowledge gaps in schistosomiasis research and development in the African region of the World Health Organization [31]. Mbugi et al. (2024) analyzed the prevalence of human schistosomiasis across various regions of mainland Tanzania and Zanzibar from 2013 to 2023 [32]. Nisa et al. (2023) utilized ecological niche models to simulate the historical distribution of schistosomiasis-transmitting snails in South Africa [33]. These studies by African scholars not only reveal the complexity of schistosomiasis transmission but also provide significant insights for future research directions, especially in the context of global climate change and human activities.

Other scholars’ research also has certain shortcomings. These are mainly reflected in three aspects. First, there is a lack of comprehensive comparison of preventive measures. Previous studies have made some progress in dealing with the transmission and treatment of schistosomiasis, but they lack comparative analysis of the effectiveness and feasibility of different prevention measures (such as health education promotion, public health facility construction, and regular screening) [34]. For example, although some studies indicate that improving health knowledge can reduce the incidence of schistosomiasis, they do not clarify its advantages and limitations compared to other prevention measures [35].

Second, there is insufficient consideration of region-specific factors. The susceptibility and transmission mechanisms of schistosomiasis may vary across different regions, yet existing research often neglects the adaptability and effectiveness of implementing preventive measures within the specific cultural, economic, and social contexts of a region. For example, certain areas in Africa might lack adequate public health facilities or face a shortage of educational resources, necessitating more region-specific studies to formulate targeted management strategies.

Third, there is a deficiency in research on preventive education and community involvement. In certain African communities, people’s awareness and reactions to schistosomiasis might be influenced by their cultural practices [5]. Research often lacks discussion on how to enhance community residents’ awareness of the disease, prevention consciousness, and active participation through education. Community-based intervention strategies may offer more viable solutions for effective prevention.

To address the aforementioned deficiencies, this paper’s contributions and innovations are manifested in three main aspects. First, conducting comparative studies. Design and implement research to compare the effects of different prevention measures (health education dissemination, construction of public health facilities, regular screening) among African children. The study should consider the feasibility and efficiency in different regional backgrounds to determine which measures or combinations thereof are most effective. Second, in-depth research into region-specific factors. Select specific African regions as the subjects of study to deeply understand how social, cultural, economic, and environmental factors within these regions affect schistosomiasis prevention strategies [9]. Analyze using game models and numerical simulations to provide a basis for formulating targeted management strategies. Third, strengthening community participation and education. Explore and implement community-based education programs, evaluating how these programs enhance awareness, prevention consciousness, and participation in schistosomiasis. The research should include innovative methods of educational content and ways to utilize community leaders and school resources to enhance information dissemination.

This study holds significant importance, which can be summarized in the following aspects. Firstly, the prevention of schistosomiasis requires systematic and multi-dimensional management strategies, including the dissemination of health education, construction of public health facilities, and implementation of regular screenings [10]. Comparative studies of different prevention methods can offer more effective schistosomiasis management and prevention programs customized for African regions. Secondly, effective schistosomiasis control strategies can reduce the disease burden and improve the quality of life for African children, thereby promoting the overall well-being of society and sustainable economic development. The dissemination of health education, improvement of public health facilities, and implementation of regular screenings can not only control the spread of schistosomiasis but also raise public awareness of the prevention of other infectious diseases, strengthening the entire community’s health management capacity. Lastly, from a management perspective, investigating schistosomiasis prevention measures can provide valuable experiences for managing other infectious diseases. Establishing an effective disease surveillance and prevention system through interdisciplinary and multi-sectoral cooperation can not only address the current schistosomiasis challenge but also prepare for future public health crises. In summary, researching how to more effectively prevent schistosomiasis among African children has direct significance for improving child health and also has a profound impact on strengthening public health management and promoting comprehensive economic and social development.

This paper refers to the experience of the governance of other infectious diseases, and compares and analyzes the three modes of popularizing health education, building public health facilities or regular screening, and derives the scope of use of different prevention modes. At the same time, because the governance of schistosomiasis in African children is continuous and dynamic, this paper uses the differential game to analyze, respectively, to establish the differential game model of these three modes, to find out the equilibrium social benefits.

## 2. Methodology

### 2.1 Problem description, hypothesis, and variable definition

#### 2.1.1 Problem description.

According to the Global Burden of Disease Study (GBD), schistosomiasis was responsible for 12,900 deaths [36]. The prevention of schistosomiasis among African children is both a health and social issue, requiring collaborative efforts from the government and societal stakeholders. As the primary provider and manager of health services, the government is responsible for delivering essential health facilities, safeguarding public health, and driving the formulation and implementation of health policies. Social forces, including non-governmental organizations, enterprises, and schools, can engage more directly with communities, provide financial or material support, and conduct localized prevention and control activities. The roles of the government and social forces in schistosomiasis prevention can be viewed as a “game,” as they strategically address healthcare needs, resource allocation, and feasible solutions. This game emphasizes collaboration over competition, aiming to resolve the health crisis collectively. The selection of “players” is not to foster conflict but to clarify their respective roles and responsibilities in maximizing public interest. Continuous cooperation between the government and social forces can be achieved through the following measures: First, policy formulation and implementation. Governments should develop targeted public health policies, such as enhancing surveillance in high-risk areas, providing drug assistance, and improving infrastructure, while ensuring their effective execution [2]. Second, resource integration. Governments and social forces can pool resources and expertise for joint prevention efforts, such as funding NGOs to leverage their professional knowledge and community connections. Third, information sharing. Strengthening communication and sharing of epidemic data, prevention experiences, and research findings, for instance, through the establishment of a schistosomiasis information database. Fourth, education and training. Jointly organizing training programs to enhance the knowledge and skills of healthcare workers and community residents, such as regular professional training or school-based health education. Fifth, leveraging social influence. Social forces can utilize media and online platforms to raise public awareness of schistosomiasis control, while businesses can contribute resources through corporate social responsibility initiatives. Through such collaboration, a synergistic effect can be achieved, effectively preventing and controlling schistosomiasis among African children and fostering a healthier environment for their development.

The sustained efforts of governments and social forces in preventing schistosomiasis among African children are driven by several key factors. First, the transmission route is complex, involving multiple factors such as water, hosts, and the environment, necessitating long-term interventions to disrupt the cycle [3]. Second, high infection rates in endemic areas, particularly among children, require continuous prevention efforts to avoid resurgence. Third, socio-economic conditions in many high-incidence African countries remain underdeveloped, necessitating ongoing investments in sanitation, infrastructure, and economic growth to reduce infection risks [37]. Fourth, health education and public awareness campaigns are essential but require sustained efforts to shift traditional behaviors and habits. Fifth, continuous monitoring and evaluation are critical to ensure the effectiveness and sustainability of prevention measures, enabling timely adjustments. Sixth, international cooperation is vital, as schistosomiasis is a global public health issue, requiring ongoing technical exchanges, financial support, and institutional innovation [38]. In summary, governments and social forces must maintain their roles and synergies to ensure the successful implementation of schistosomiasis prevention measures.

Specifically, in order to effectively prevent schistosomiasis in African children, government and social forces mainly have three prevention modes.

(1) Popularizing health education mode. To effectively prevent schistosomiasis among African children, governments and social forces can implement the following measures to promote universal health education. First, develop a comprehensive publicity plan, including material design, content formulation, and communication channels. Second, create accessible publicity materials detailing schistosomiasis transmission routes, prevention methods, symptoms, and treatment, using local languages and easy-to-understand formats like cartoons and illustrations. Third, integrate schistosomiasis prevention knowledge into school curricula to educate children early [20]. Fourth, organize public welfare activities, such as lectures, free clinics, and exhibitions, to disseminate knowledge widely. Fifth, utilize media platforms, including radio, television, newspapers, and the internet, to raise awareness of schistosomiasis risks and prevention [39]. Sixth, collaborate with local communities, village committees, and religious groups to enhance residents’ understanding of prevention and treatment. These strategies can effectively popularize health education, increase awareness, and reduce schistosomiasis infection risks among African children [18].
(2) Public health facilities construction mode. Governments and social forces can implement the following strategies in public health infrastructure development to effectively prevent schistosomiasis among African children. First, establish safe water supply systems, such as deep wells, rainwater harvesting, and purification equipment, to minimize children’s exposure to contaminated water [23]. Safe water supplies radically reduce parasitic infection among South African children. Second, construct and maintain clean public toilets to prevent fecal transmission of schistosomiasis. Third, develop sewage treatment facilities to ensure proper waste and fluid disposal, preventing contamination of water bodies [4]. Fourth, improve housing conditions by addressing insect and moisture control and ensuring proper ventilation to eliminate breeding grounds for schistosomiasis. Fifth, establish and maintain community medical facilities, particularly for schistosomiasis treatment, to enable timely intervention and reduce pathogen transmission. These efforts require collaboration between governments, communities, and organizations to build and sustain public health infrastructure, thereby addressing schistosomiasis at its source.
(3) Regular screening mode. According to the WHO Africa Report, in 2023, the total number of people requiring preventive chemotherapy globally is 253.8 million, 135 million of whom are school-age children [1]. Regular screening can provide assurance for preventive chemotherapy. Governments and social forces can implement the following measures for regular screening to effectively prevent schistosomiasis among African children. First, develop a detailed screening plan, specifying timing, locations, target populations, and methods to ensure organized execution [27]. Second, foster cooperation with schools, community health centers, and medical institutions to facilitate screening activities. Third, promote awareness through media channels such as radio, television, newspapers, and community bulletin boards to inform the public about screening schedules and procedures. Fourth, conduct regular school-based screenings, including physical exams and tests, to identify and treat infected students promptly. Fifth, organize community screenings through health centers or free clinic events to detect and manage potential cases. Sixth, offer convenient screening services, such as door-to-door visits in remote areas, to maximize participation. These measures enable governments and social forces to effectively detect and treat schistosomiasis in African children, reducing transmission risks and safeguarding their health.

**Fig 1:**
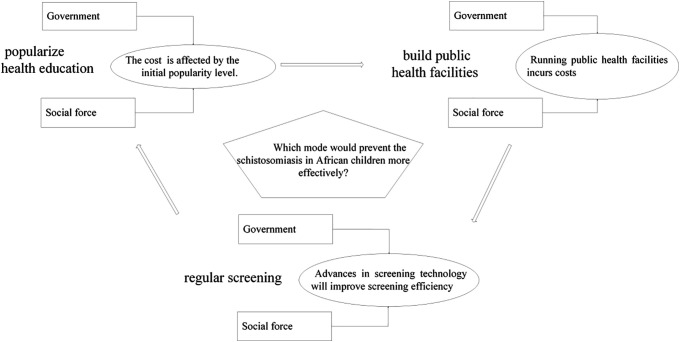
Relationship between these three modes for preventing the schistosomiasis.

The relationship between the three modes for preventing the schistosomiasis in African children is shown in Fig 1.

#### 2.1.2 Hypothesis.

(1) The government’s cost of popularizing health education is affected by the initial popularity level.

When health education is popularized in a society, the cost of resources, time and money required to popularize health education will be relatively low, because many basic knowledge has been widely disseminated and the public has a certain degree of health awareness. On this basis, the government can carry out deeper and more targeted health education work. On the contrary, if a society has a low popularity of health education in the initial state, the cost of resources, time and money required by the government to popularize health education will increase accordingly. Therefore, the government needs to strengthen the promotion of basic health knowledge, improve the public’s health awareness and behavior level, and try to overcome possible cultural, social and economic obstacles [39]. In summary, when promoting health education, the government needs to develop appropriate policies and strategies according to the initial popularity of the society, and on this basis, increase investment to improve the popularity of health education, in order to achieve the goal of improving the overall health level of the public [18]. At the same time, cooperation and synergy are also key, including the participation and efforts of social organizations, schools, enterprises and the public themselves.

Real-world examples also validate this hypothesis. In some African countries with high malaria prevalence, governments have found that the cost of promoting the use of mosquito nets and anti-malarial drugs is relatively lower in communities that already have a certain foundation of health education (e.g., understanding the transmission routes and preventive measures of malaria) [40]. This is because residents in these areas have a higher acceptance of health education, and the government only needs to provide supplementary education and resource allocation. In contrast, in areas with weak health education foundations, the government needs to invest more resources in basic education and promotion, significantly increasing the costs.

In South Africa, during the initial promotion of HIV/AIDS health education programs, it was observed that in areas where residents had already been exposed to HIV/AIDS knowledge through non-governmental organizations or community projects, the subsequent promotion costs for the government were lower [41]. However, in remote areas where such knowledge had never been introduced, the government had to start from scratch, including training educators and producing promotional materials, leading to a significant increase in costs. These cases collectively validate the hypothesis.

(2) Running public health facilities incurs costs.

There are some costs associated with running public health facilities. These costs include but are not limited to the following: First, facility construction and maintenance. The construction of public health facilities requires some initial investment, including land purchase, construction, equipment procurement and other costs [34]. In addition, regular maintenance and repair work will also incur some costs. Second, operation management. The operation of public health facilities involves a wide range of management activities, including the daily management of the facilities and the recruitment and training of personnel [42]. In addition, there are also operational costs to consider, such as the wages of security guards, cleaners and other service personnel. Third, water and electricity costs. In the process of using public health facilities, water and electricity resources are consumed, so water and electricity costs are also part of the fixed costs in the operation process. Fourth, health education and public activities. Many public health facilities will hold regular educational activities and public activities to promote public health awareness and behavior, which also requires budget and investment [34]. Fifth, law and safety. In order to avoid legal and safety risks, it is necessary to comply with local and national regulations, safety and health standards, etc. It is worth mentioning that although there is a certain cost to operate public health facilities, such investment helps to improve public health, prevent the spread of diseases, and enhance the health and well-being of the community. Therefore, such investment has its value and returns in the long run.

Real-world examples also support this hypothesis. In Ethiopia, the government and non-governmental organizations have collaborated to establish numerous rural health stations aimed at providing basic medical services. The operational costs of these health stations include the maintenance of medical equipment, procurement of medicines, salaries of medical staff, and daily expenses such as utilities. Due to insufficient funding, some health stations face issues such as aging equipment, shortages of medicines, and staff attrition, leading to a decline in service quality [43].

(3) Advances in screening technology will improve screening efficiency.

In high transmission settings, where mothers bathe their children daily with water extracted from the environment, many infants and preschool children suffer from schistosomiasis [19]. To prevent the transmission of schistosomiasis, children need to be screened. In medicine and health, screening refers to the initial examination of a group of asymptomatic individuals to identify those who are ill or at high risk. With the development of science and technology, screening technology has been improved as follows. First, improved accuracy. New screening technologies have higher sensitivity and specificity than traditional methods, which means lower misdiagnosis and missed diagnosis rates and more accurate identification of patients [6]. Second, shorter detection time. Technological advances have made the screening process more rapid and efficient. The optimization of detection methods can reduce the time spent in the operation and shorten the waiting time for results [44]. Third, more convenient self-testing. Some screening technologies allow patients to perform simple tests at home, such as blood glucose tests for diabetics and home pregnancy tests for pregnant women. Fourth, non-invasive detection. Some technological advances have reduced the invasiveness of the screening process. For example, non-invasive prenatal screening can detect chromosomal abnormalities in the fetus by taking blood from the pregnant woman without the need for invasive puncture. Fifth, reduced costs. As the technology matures and production increases, the cost of the test may decrease, making it accessible to more people. Sixth, the application of big data and artificial intelligence. Technological progress allows big data and artificial intelligence to play an important role in screening, which can greatly improve the efficiency and accuracy of the screening process. In summary, advances in screening technology can indeed improve the efficiency of screening, which helps to detect and diagnose diseases earlier, provide patients with more timely and accurate treatment options, and thus improve the prognosis and quality of life of patients.

Real-world examples also validate this hypothesis. Traditional HIV screening methods primarily rely on enzyme-linked immunosorbent assay (ELISA), which requires laboratory equipment and trained technicians to operate, and results may take a considerable amount of time to obtain. With the introduction of rapid test kits, the efficiency and convenience of HIV screening have significantly improved. For instance, in Kenya, the introduction of rapid test kits has greatly enhanced screening efficiency, enabling coverage of a larger population in a shorter time frame, thereby facilitating earlier detection and treatment of HIV [45].

Traditional cervical cancer screening methods primarily rely on Pap smear tests, which require specialized technicians for analysis and may carry certain margins of error. With the introduction of liquid-based cytology (LBC) and HPV DNA testing technologies, the efficiency and accuracy of cervical cancer screening have significantly improved. For example, in Rwanda, the introduction of HPV DNA testing has greatly enhanced screening efficiency, enabling coverage of a larger population in a shorter time frame, thereby facilitating earlier detection and treatment of cervical cancer [46]. These cases collectively validate the hypothesis.

#### 2.1.3 Variable definition.

When constructing the differential game model in this article, many parameters and variables are designed. These parameters and variables are defined as shown in Table 1.

**Table 1:** The main definition of variables and parameters in this article.

variables and parameters	specific meaning
*Y=*{*P,B,R*}	three prevention modes for schistosomiasis in African children (popularize health education, build public health facilities or regular screening)
independent variable	
*F*_*Y*1_(*t*)	the extent of the government’s efforts to prevent schistosomiasis in African children under mode *Y*
*F*_*Y*2_(*t*)	the extent of the social force’s efforts to prevent schistosomiasis in African children under mode *Y*
*x*_*Y*1_(*t*)	the government’s reputation for preventing schistosomiasis in African children under mode *Y*
*x*_*Y*2_(*t*)	the social force’s reputation for preventing schistosomiasis in African children under mode *Y*
parameter	
*ρ*	the discount rate that occurs over time, 0 ≤ *ρ* ≤ 1
*δ*	decay of reputation, *δ* > 0
*a*_1_, *a*_2_	the benefits that government or social force gain by preventing schistosomiasis in African children at a unit level, *a*_1_, *a*_2_ > 0
*c*_1_,*c*_2_	the cost to government or social force of preventing schistosomiasis in African children at a unit level, *c*_1_, *c*_2_ > 0
*c*_*B*_	cost per unit of public health facility operation, *c*_*B*_ > 0
*β*	prevalence of schistosomiasis, *β* > 0
*l*	the positive effects of reputation, *l* > 0
*f*_*P*_	the reputation gained for promoting health literacy, *f*_*P*_ > 0
*f*_*B*_	the reputation generated by the number of units of public health facilities operating, *f*_*B*_ > 0
*T*	the level of development of screening technology, *T* > 0
*f*_*R*_	the level of discontent caused by screening. Because screening takes time, it can lead to public discontent, *f*_*R*_ > 0
*f*_1_,*f*_2_	the reputation gained by government or social force for preventing schistosomiasis in African children at a unit level, *f*_1_,*f*_2_ > 0
function	
*J*_*Y*1_(*t*)	the social welfare function of government under the mode *Y*
*J*_*Y*2_(*t*)	the social welfare function of social force under the mode *Y*
*V*_*Y*1_(*t*)	the social benefits of government under the mode *Y*
*V*_*Y*2_(*t*)	the social benefits of social force under the mode *Y*

In Table 1, the government and social forces, as game players, exhibit significant differences in their focus and objectives regarding social benefits. For the government, its social benefits are primarily reflected in the widespread implementation and long-term effects of public health policies. For instance, through the promotion of health education, the construction of public health facilities, or regular screenings, the government aims to reduce the incidence of schistosomiasis among African children, thereby improving overall population health, reducing healthcare expenditures, and fostering social stability and economic development [47]. In contrast, the social benefits of social forces are more focused on direct participation and community-level impacts. For example, through the efforts of non-governmental organizations, community volunteers, or educational institutions, social forces strive to promote the dissemination of health education, enhance public awareness of disease prevention, and respond to and address health issues rapidly through community mobilization and resource integration. In short, the government’s social benefits are more macro and systematic, while the social benefits of social forces are more specific and flexible, emphasizing grassroots actions and community engagement.

In the differential game model, *F*_*Y*1_(*t*) and *F*_*Y*2_(*t*) represent the efforts of the government and social forces in preventing schistosomiasis among African children, respectively. The primary difference between these two variables lies in their respective entities and resource allocation methods. *F*_*Y*1_(*t*) reflects the government’s resource investment, policy implementation intensity, and direct intervention measures under a specific mode (mode *Y*), such as fiscal allocations, infrastructure development, or the promotion of health education programs. In contrast, *F*_*Y*2_(*t*) represents the efforts of social forces (e.g., non-governmental organizations, community groups, or private institutions) under the same mode, which may include financial donations, volunteer activities, or community mobilization. Government efforts tend to be more systematic and mandatory, while those of social forces are often more flexible and targeted, though both share the common goal of disease prevention.

On the other hand, *x*_*Y*1_(*t*) and *x*_*Y*2_(*t*) represent the reputations of the government and social forces in preventing schistosomiasis among African children, respectively. The distinction between these two variables lies in the public perception and trust in the respective entities. *x*_*Y*1_(*t*) reflects the public recognition and trust in the government’s implementation of preventive measures under the *Y* model, such as policy transparency, execution effectiveness, and responsiveness to public needs. In contrast, *x*_*Y*2_(*t*) measures the reputation of social forces under the same model, potentially based on the efficacy of their programs, collaboration with communities, and support for vulnerable groups. The government’s reputation is typically linked to the long-term effects and stability of policies, while the reputation of social forces may rely more on the short-term outcomes of specific projects and public sentiment. Enhancing reputation can further motivate entities to intensify their efforts in disease prevention, creating a virtuous cycle.

In the differential game model, the parameters *ρ* (the discount rate over time) and *δ* (the reputation decay rate) reflect the influence of time factors on decision-making and reputation dynamics. The value of *ρ* ranges from 0 to 1, measuring the present value of future benefits or costs—the smaller the value, the more the decision-maker prioritizes short-term gains over long-term effects. Meanwhile, *δ* describes the natural rate at which reputation decays over time; a higher value indicates that sustaining reputation requires greater continuous effort. Together, these two parameters shape the resource allocation and long-term planning of governments and social forces in strategies to prevent schistosomiasis.

Parameters *a*_1_ and *a*_2_ respectively represent the benefits obtained by the government and social forces in preventing schistosomiasis in African children at the unit level, while *c*_1_ and *c*_2_ reflect the costs of implementing prevention measures at the unit level. These parameters are used to measure the input-output ratio of different entities in prevention strategies, in order to optimize resource allocation. In addition, *c*_*B*_ (cost per unit of public health facility operation) and *β* (prevalence of schistosomiasis) further quantified the impact of public health facility construction and disease transmission on strategy effectiveness. These parameters collectively determine the economic feasibility and actual effectiveness of preventive measures.

The positive impact of reputation and the reputation gained by government or social forces in preventing schistosomiasis in African children at the unit level describe the motivating effect of reputation on subject behavior. *l* measures the positive impact of reputation enhancement on the level of effort of the subject, while *f*_1_ and *f*_2_ respectively quantify the reputation benefits obtained by the government and social forces through preventive measures. In addition, the level of development of screening technology and the degree of dissatisfaction caused by screening reflect the balance between the technological progress of screening strategies and public acceptance. These parameters work together to provide a comprehensive analytical framework for the model to evaluate the overall effectiveness of different prevention strategies.

### 2.2 Differential game of three prevention modes

Differential games is a mathematical theory that combines the concepts of differential equations and game theory to understand strategic interactions in decision-making processes that can be represented by dynamic systems, often described by differential equations. In differential games, the decisions of participants (called players) change continuously over time, and the decisions of each player affect the utility or benefits of the other players. Differential games are often used to analyze competition and cooperation problems in fields such as economics, military, or environmental policy [40]. A typical feature of differential games is dynamic strategies that advance over time, considering possible future states and possible actions of opponents. In addition, equilibrium concepts of differential games, such as Nash equilibrium, are often used to analyze stable strategy configurations in dynamic situations. In the analysis of differential games, strategies that are in some sense optimal are usually found, these strategies are called feedback Nash equilibrium or open-loop Nash equilibrium. The basic mathematical tools of differential games include dynamic optimization, differential equations, optimal control methods in control theory, and so on. By solving the optimal control problem in a dynamic system to find an equilibrium strategy, concrete conclusions can be drawn about how players adjust their strategies in response to opponents’ actions and system evolution.

Refer to the relevant differential game models [48,49], if the government and social force prevent schistosomiasis in African children through the mode of popularizing health education, then their social welfare function can be expressed as:


$$J_{P1}=\int_{\text{0}}^{\infty }[a_{\text{1}}F_{P1}\left(t\right)-\frac{c_{\text{1}}}{2ln\left(\text{e+}\beta \right)}\overset{\text{2}}{\underset{P1}{F}}\left(t\right)+lx_{P1}\left(t\right)]\text{\textbackslash{}hspace\{0.33em}\}e^{-\rho t}dt$$ (1)

$$J_{P2}=\int_{\text{0}}^{\infty }[a_{\text{2}}F_{P2}\left(t\right)-\frac{c_{\text{2}}}{\text{2}}\overset{\text{2}}{\underset{P2}{F}}\left(t\right)+lx_{P2}\left(t\right)]\text{\textbackslash{}hspace\{0.33em}\}e^{-\rho t}dt$$ (2)

In the above formulae, $a_{\text{1}}F_{P1}\left(t\right)$ represents the government’s benefit from schistosomiasis prevention. $\frac{c_{\text{1}}}{2ln\left(\text{e+}\beta \right)}\overset{\text{2}}{\underset{P1}{F}}\left(t\right)$ represents the government’s cost of schistosomiasis prevention. ln(e+β) represents the impact of coverage on the cost of coverage. $lx_{P1}\left(t\right)$ represents the positive impact of reputation on the government’s benefit. $a_{\text{2}}F_{P2}\left(t\right)$ represents the social force’s benefit from schistosomiasis prevention. $\frac{c_{\text{2}}}{\text{2}}\overset{\text{2}}{\underset{P2}{F}}\left(t\right)$ represents the social force’s cost of schistosomiasis prevention. $lx_{P2}\left(t\right)$ represents the positive impact of reputation on the social force’s benefit.

The change in the reputation of government and social force under the mode of popularizing health education can be expressed as:


$${\dot{x}}_{P1}\left(t\right)=\left(f_{\text{1}}+f_{P}\right)F_{P1}\left(t\right)-\delta x_{P1}\left(t\right)$$ (3)

$${\dot{x}}_{P2}\left(t\right)=\left(f_{\text{2}}+f_{P}\right)F_{P2}\left(t\right)-\delta x_{P2}\left(t\right)$$ (4)

In the above formulae, $f_{\text{1}}F_{P1}\left(t\right)$ means the increase in the reputation of the government by preventing schistosomiasis. $f_{P}F_{P1}\left(t\right)$ means the gain in the reputation of the government by popularizing health knowledge. $\delta x_{P1}\left(t\right)$ means the decrease in the reputation of the government. $f_{\text{2}}F_{P2}\left(t\right)$ means the increase in the reputation of the social forces by preventing schistosomiasis. $f_{P}F_{P2}\left(t\right)$ means the gain in the reputation of the social forces by popularizing health knowledge. $\delta x_{P2}\left(t\right)$ means the decrease in the reputation of the social forces.

If the government and social force prevent schistosomiasis in African children through the mode of public health facilities construction, then their social welfare function can be expressed as:


$$J_{B1}=\int_{\text{0}}^{\infty }[a_{\text{1}}F_{B1}\left(t\right)-\frac{\left(c_{\text{1}}+c_{B}\right)}{\text{2}}\overset{\text{2}}{\underset{B1}{F}}\left(t\right)+lx_{B1}\left(t\right)]\text{\textbackslash{}hspace\{0.33em}\}e^{-\rho t}dt$$ (5)

$$J_{B2}=\int_{\text{0}}^{\infty }[a_{\text{2}}F_{B2}\left(t\right)-\frac{c_{\text{2}}}{\text{2}}\overset{\text{2}}{\underset{B2}{F}}\left(t\right)+lx_{B2}\left(t\right)]\text{\textbackslash{}hspace\{0.33em}\}e^{-\rho t}dt$$ (6)

In the above formulae, $a_{\text{1}}F_{B1}\left(t\right)$ represents the benefit gained by the government from schistosomiasis prevention. $\frac{c_{\text{1}}}{\text{2}}\overset{\text{2}}{\underset{B1}{F}}\left(t\right)$ represents the cost paid by the government for schistosomiasis prevention. $\frac{c_{B}}{\text{2}}\overset{\text{2}}{\underset{B1}{F}}\left(t\right)$ represents the cost of public health facilities. $lx_{B1}\left(t\right)$ represents the positive impact of reputation on the government’s efficiency. $a_{\text{2}}F_{B2}\left(t\right)$ represents the benefit gained by the social force from schistosomiasis prevention. $\frac{c_{\text{2}}}{\text{2}}\overset{\text{2}}{\underset{B2}{F}}\left(t\right)$ represents the cost paid by the social force for schistosomiasis prevention. $lx_{B2}\left(t\right)$ represents the positive impact of reputation on the social force’s efficiency.

The change in the reputation of government and social force under the mode of public health facilities construction can be expressed as:


$${\dot{x}}_{B1}\left(t\right)=\left(f_{\text{1}}+f_{B}\right)F_{B1}\left(t\right)-\delta x_{B1}\left(t\right)$$ (7)

$${\dot{x}}_{B2}\left(t\right)=f_{\text{2}}F_{B2}\left(t\right)-\delta x_{B2}\left(t\right)$$ (8)

In the above formulae, $f_{\text{1}}F_{B1}\left(t\right)$ represents the reputation of the government for schistosomiasis prevention. $f_{B}F_{B1}\left(t\right)$ represents the reputation of the public health facilities. $\delta x_{B1}\left(t\right)$ represents the decline of the government’s reputation. $f_{\text{2}}F_{B2}\left(t\right)$ represents the reputation of the social forces for schistosomiasis prevention. $\delta x_{B2}\left(t\right)$ represents the decline of the social forces’ reputation.

If the government and social force prevent schistosomiasis in African children through the mode of regular screening, then their social welfare function can be expressed as:


$$J_{R1}=\int_{\text{0}}^{\infty }[a_{\text{1}}ln\left(1\text{+}T\right)F_{R1}\left(t\right)-\frac{c_{\text{1}}}{\text{2}}\overset{\text{2}}{\underset{R1}{F}}\left(t\right)+lx_{R1}\left(t\right)]\text{\textbackslash{}hspace\{0.33em}\}e^{-\rho t}dt$$ (9)

$$J_{R2}=\int_{\text{0}}^{\infty }[a_{\text{2}}ln\left(1\text{+}T\right)F_{R2}\left(t\right)-\frac{c_{\text{2}}}{\text{2}}\overset{\text{2}}{\underset{R2}{F}}\left(t\right)+lx_{R2}\left(t\right)]\text{\textbackslash{}hspace\{0.33em}\}e^{-\rho t}dt$$ (10)

In the above formulae, $a_{\text{1}}ln\left(1\text{+}T\right)F_{R1}\left(t\right)$ represents the government’s benefit from schistosomiasis prevention. $\frac{c_{\text{1}}}{\text{2}}\overset{\text{2}}{\underset{R1}{F}}\left(t\right)$ represents the government’s cost of schistosomiasis prevention. ln(1+T) represents the impact of screening technology on screening efficiency. $lx_{R1}\left(t\right)$ represents the positive impact of reputation on government’s benefit. $a_{\text{2}}ln\left(1\text{+}T\right)F_{R2}\left(t\right)$ represents the social force’s benefit from schistosomiasis prevention. $\frac{c_{\text{2}}}{\text{2}}\overset{\text{2}}{\underset{R2}{F}}\left(t\right)$ represents the social force’s cost of schistosomiasis prevention. $lx_{R2}\left(t\right)$ represents the positive impact of reputation on social force’s benefit.

The change in the reputation of government and social force under the mode of regular screening can be expressed as:


$${\dot{x}}_{R1}\left(t\right)=\left(f_{\text{1}}-f_{R}\right)F_{R1}\left(t\right)-\delta x_{R1}\left(t\right)$$ (11)

$${\dot{x}}_{R2}\left(t\right)=\left(f_{\text{2}}-f_{R}\right)F_{R2}\left(t\right)-\delta x_{R2}\left(t\right)$$ (12)

In the above formulae, $f_{\text{1}}F_{R1}\left(t\right)$ represents the government’s reputation for preventing schistosomiasis. $f_{R}F_{R1}\left(t\right)$ represents the public’s dissatisfaction with the government due to the time taken by screening. $\delta x_{R1}\left(t\right)$ represents the government’s reputation declining. $f_{\text{2}}F_{R2}\left(t\right)$ represents the social force’s reputation for preventing schistosomiasis. $f_{R}F_{R2}\left(t\right)$ represents the public’s dissatisfaction with the social force due to the time taken by screening. $\delta x_{R2}\left(t\right)$ represents the social force’s reputation declining.

## 3. Results

In the differential game, the government and social force in the process of preventing schistosomiasis in African children are not only affected by control variables and parameters, but also change over time. In order to better calculate the control benefits and social benefits, the HJB formula is used. The HJB formula is a partial differential equation, which is the core of optimal control.

### 3.1 HJB formula

Under the mode of popularizing health education, the HJB equation of the social welfare function of the government and social force are:


$$\rho V_{P1}=\underset{F_{P1}\left(t\right)}{max}\{[a_{\text{1}}F_{P1}\left(t\right)-\frac{c_{\text{1}}}{2ln\left(\text{e+}\beta \right)}\overset{\text{2}}{\underset{P1}{F}}\left(t\right)+lx_{P1}\left(t\right)]+\frac{\partial V_{P1}}{\partial x_{P1}}\left[\left(f_{\text{1}}+f_{P}\right)F_{P1}\left(t\right)-\delta x_{P1}\left(t\right)\right]\}$$ (13)

$$\rho V_{P2}=\underset{F_{P2}\left(t\right)}{max}\{[a_{\text{2}}F_{P2}\left(t\right)-\frac{c_{\text{2}}}{\text{2}}\overset{\text{2}}{\underset{P2}{F}}\left(t\right)+lx_{P2}\left(t\right)]+\frac{\partial V_{P2}}{\partial x_{P2}}\left[\left(f_{\text{2}}+f_{P}\right)F_{P2}\left(t\right)-\delta x_{P2}\left(t\right)\right]\}$$ (14)

Under the mode of public health facilities construction, the HJB equation of the social welfare function of the government and social force are:


$$\rho V_{B1}=\underset{F_{B1}\left(t\right)}{max}\{[a_{\text{1}}F_{B1}\left(t\right)-\frac{\left(c_{\text{1}}+c_{B}\right)}{\text{2}}\overset{\text{2}}{\underset{B1}{F}}\left(t\right)+lx_{B1}\left(t\right)]+\frac{\partial V_{B1}}{\partial x_{B1}}\left[\left(f_{\text{1}}+f_{B}\right)F_{B1}\left(t\right)-\delta x_{B1}\left(t\right)\right]\}$$ (15)

$$\rho V_{B2}=\underset{F_{B2}\left(t\right)}{max}\{[a_{\text{2}}F_{B2}\left(t\right)-\frac{c_{\text{2}}}{\text{2}}\overset{\text{2}}{\underset{B2}{F}}\left(t\right)+lx_{B2}\left(t\right)]+\frac{\partial V_{B2}}{\partial x_{B2}}\left[f_{\text{2}}F_{B2}\left(t\right)-\delta x_{B2}\left(t\right)\right]\}$$ (16)

Under the mode of regular screening, the HJB equation of the social welfare function of the government and social force are:


$$\rho V_{R1}=\underset{F_{R1}\left(t\right)}{max}\{[a_{\text{1}}ln\left(1\text{+}T\right)F_{R1}\left(t\right)-\frac{c_{\text{1}}}{\text{2}}\overset{\text{2}}{\underset{R1}{F}}\left(t\right)+lx_{R1}\left(t\right)]+\frac{\partial V_{R1}}{\partial x_{R1}}\left[\left(f_{\text{1}}-f_{R}\right)F_{R1}\left(t\right)-\delta x_{R1}\left(t\right)\right]\}$$ (17)

$$\rho V_{R2}=\underset{F_{R2}\left(t\right)}{max}\{[a_{\text{2}}ln\left(1\text{+}T\right)F_{R2}\left(t\right)-\frac{c_{\text{2}}}{\text{2}}\overset{\text{2}}{\underset{R2}{F}}\left(t\right)+lx_{R2}\left(t\right)]+\frac{\partial V_{R2}}{\partial x_{R2}}\left[\left(f_{\text{2}}-f_{R}\right)F_{R2}\left(t\right)-\delta x_{R2}\left(t\right)\right]\}$$ (18)

### 3.2 Result of equilibrium

Proposition 1: Under the mode of popularizing health education, the extent of efforts to prevent schistosomiasis in African children and social benefits of government and social force are respectively (the specific solving procedure is shown in S1 File):


$$\overset{*}{\underset{P1}{F}}\left(t\right)=\frac{a_{\text{1}}}{c_{\text{1}}}ln\left(\text{e+}\beta \right)+\frac{f_{\text{1}}+f_{P}}{c_{\text{1}}}\frac{l}{\rho +\delta }ln\left(\text{e+}\beta \right)$$ (19)

$$\overset{*}{\underset{P2}{F}}\left(t\right)=\frac{a_{\text{2}}}{c_{\text{2}}}+\frac{f_{\text{2}}+f_{P}}{c_{\text{2}}}\frac{l}{\rho +\delta }$$ (20)

$$\begin{array}{c} \overset{*}{\underset{P1}{V}}=\frac{\text{1}}{\rho }a_{\text{1}}\left[\frac{a_{\text{1}}}{c_{\text{1}}}ln\left(\text{e+}\beta \right)+\frac{f_{\text{1}}+f_{P}}{c_{\text{1}}}\frac{l}{\rho +\delta }ln\left(\text{e+}\beta \right)\right]-{\left[\frac{a_{\text{1}}}{c_{\text{1}}}ln\left(\text{e+}\beta \right)+\frac{f_{\text{1}}+f_{P}}{c_{\text{1}}}\frac{l}{\rho +\delta }ln\left(\text{e+}\beta \right)\right]}^{\text{2}}\\ \text{\textbackslash{}hspace\{0.33em}\;\;\;\;\;\;\}\frac{c_{\text{1}}}{2ln\left(\text{e+}\beta \right)}\frac{\text{1}}{\rho }+\frac{\text{1}}{\rho }\frac{l}{\rho +\delta }\left(f_{\text{1}}+f_{P}\right)\left[\frac{a_{\text{1}}}{c_{\text{1}}}ln\left(\text{e+}\beta \right)+\frac{f_{\text{1}}+f_{P}}{c_{\text{1}}}\frac{l}{\rho +\delta }ln\left(\text{e+}\beta \right)\right]+\frac{l}{\rho +\delta }x_{P1} \end{array}$$ (21)

$$\begin{array}{c} \overset{\text{*}}{\underset{P2}{V}}=\frac{l}{\rho +\delta }x_{P2}+\frac{\text{1}}{\rho }a_{\text{2}}\left(\frac{a_{\text{2}}}{c_{\text{2}}}+\frac{f_{\text{2}}+f_{P}}{c_{\text{2}}}\frac{l}{\rho +\delta }\right)-\frac{c_{\text{2}}}{\text{2}}\frac{\text{1}}{\rho }{\left(\frac{a_{\text{2}}}{c_{\text{2}}}+\frac{f_{\text{2}}+f_{P}}{c_{\text{2}}}\frac{l}{\rho +\delta }\right)}^{\text{2}}\\ \text{\textbackslash{}hspace\{0.33em}\;\;\;\;\}+\frac{l}{\rho +\delta }\frac{\text{1}}{\rho }\left(f_{\text{2}}+f_{P}\right)\left(\frac{a_{\text{2}}}{c_{\text{2}}}+\frac{f_{\text{2}}+f_{P}}{c_{\text{2}}}\frac{l}{\rho +\delta }\right) \end{array}$$ (22)

Finding 1: The greater the prevalence of schistosomiasis, the greater the efforts of governments to popularize health education. The greater the reputation for popularizing health knowledge, the greater the efforts of governments and social forces to prevent schistosomiasis in African children.

Proposition 2: Under the mode of public health facilities construction, the extent of efforts to prevent schistosomiasis in African children and social benefits of government and social force are respectively (the specific solving procedure is shown in S2 File):


$$\overset{*}{\underset{B1}{F}}\left(t\right)=\frac{a_{\text{1}}}{c_{\text{1}}+c_{B}}+\frac{f_{\text{1}}+f_{B}}{c_{\text{1}}+c_{B}}\frac{l}{\rho +\delta }$$ (23)

$$\overset{*}{\underset{B2}{F}}\left(t\right)=\frac{a_{\text{2}}}{c_{\text{2}}}+\frac{f_{\text{2}}}{c_{\text{2}}}\frac{l}{\rho +\delta }$$ (24)

$$\begin{array}{c} \overset{*}{\underset{B1}{V}}=\frac{l}{\rho +\delta }x_{B1}+\frac{\text{1}}{\rho }a_{\text{1}}\left(\frac{a_{\text{1}}}{c_{\text{1}}+c_{B}}+\frac{f_{\text{1}}+f_{B}}{c_{\text{1}}+c_{B}}\frac{l}{\rho +\delta }\right)-\frac{\left(c_{\text{1}}+c_{B}\right)}{\text{2}}\frac{\text{1}}{\rho }{\left(\frac{a_{\text{1}}}{c_{\text{1}}+c_{B}}+\frac{f_{\text{1}}+f_{B}}{c_{\text{1}}+c_{B}}\frac{l}{\rho +\delta }\right)}^{\text{2}}\\ \text{\textbackslash{}hspace\{0.33em}\;\;\;\;\;\;\}+\frac{\text{1}}{\rho }\frac{l}{\rho +\delta }\left(f_{\text{1}}+f_{B}\right)\left(\frac{a_{\text{1}}}{c_{\text{1}}+c_{B}}+\frac{f_{\text{1}}+f_{B}}{c_{\text{1}}+c_{B}}\frac{l}{\rho +\delta }\right) \end{array}$$ (25)

$$\overset{\text{*}}{\underset{B2}{V}}=\frac{l}{\rho +\delta }x_{B2}+\frac{\text{1}}{\rho }a_{\text{2}}\left(\frac{a_{\text{2}}}{c_{\text{2}}}+\frac{f_{\text{2}}}{c_{\text{2}}}\frac{l}{\rho +\delta }\right)-\frac{c_{\text{2}}}{\text{2}}\frac{\text{1}}{\rho }{\left(\frac{a_{\text{2}}}{c_{\text{2}}}+\frac{f_{\text{2}}}{c_{\text{2}}}\frac{l}{\rho +\delta }\right)}^{\text{2}}+\frac{\text{1}}{\rho }\frac{\partial V_{B2}}{\partial x_{B2}}f_{\text{2}}\left(\frac{a_{\text{2}}}{c_{\text{2}}}+\frac{f_{\text{2}}}{c_{\text{2}}}\frac{l}{\rho +\delta }\right)$$ (26)

Finding 2: The greater the cost of operating a public health facility per unit, the less the government tries to build public facilities.

Proposition 3: Under the mode of regular screening, the extent of efforts to prevent schistosomiasis in African children and social benefits of government and social force are respectively (the specific solving procedure is shown in S3 File):


$$\overset{*}{\underset{R1}{F}}\left(t\right)=\frac{a_{\text{1}}}{c_{\text{1}}}ln\left(1\text{+}T\right)+\frac{f_{\text{1}}-f_{R}}{c_{\text{1}}}\frac{l}{\rho +\delta }$$ (27)

$$\overset{*}{\underset{R2}{F}}\left(t\right)=\frac{a_{\text{2}}}{c_{\text{2}}}ln\left(1\text{+}T\right)+\frac{f_{\text{2}}-f_{R}}{c_{\text{2}}}\frac{l}{\rho +\delta }$$ (28)

$$\begin{array}{c} \overset{*}{\underset{R1}{V}}=\frac{l}{\rho +\delta }x_{R1}+\frac{\text{1}}{\rho }a_{\text{1}}ln\left(1\text{+}T\right)\left[\frac{a_{\text{1}}}{c_{\text{1}}}ln\left(1\text{+}T\right)+\frac{f_{\text{1}}-f_{R}}{c_{\text{1}}}\frac{l}{\rho +\delta }\right]-\frac{c_{\text{1}}}{\text{2}}\frac{\text{1}}{\rho }{\left[\frac{a_{\text{1}}}{c_{\text{1}}}ln\left(1\text{+}T\right)+\frac{f_{\text{1}}-f_{R}}{c_{\text{1}}}\frac{l}{\rho +\delta }\right]}^{\text{2}}\\ \text{\textbackslash{}hspace\{0.33em}\;\;\;\;\;\;\}+\frac{l}{\rho +\delta }\frac{\text{1}}{\rho }\left(f_{\text{1}}-f_{R}\right)\left[\frac{a_{\text{1}}}{c_{\text{1}}}ln\left(1\text{+}T\right)+\frac{f_{\text{1}}-f_{R}}{c_{\text{1}}}\frac{l}{\rho +\delta }\right] \end{array}$$ (29)

$$\begin{array}{c} \overset{\text{*}}{\underset{R2}{V}}=\frac{l}{\rho +\delta }x_{R2}+\frac{\text{1}}{\rho }a_{\text{2}}ln\left(1\text{+}T\right)\left[\frac{a_{\text{2}}}{c_{\text{2}}}ln\left(1\text{+}T\right)+\frac{f_{\text{2}}-f_{R}}{c_{\text{2}}}\frac{l}{\rho +\delta }\right]-\frac{c_{\text{2}}}{\text{2}}\frac{\text{1}}{\rho }{\left[\frac{a_{\text{2}}}{c_{\text{2}}}ln\left(1\text{+}T\right)+\frac{f_{\text{2}}-f_{R}}{c_{\text{2}}}\frac{l}{\rho +\delta }\right]}^{\text{2}}\\ \text{\textbackslash{}hspace\{0.33em}\;\;\;\;\;\;\;\;\}+\frac{l}{\rho +\delta }\left(f_{\text{2}}-f_{R}\right)\frac{\text{1}}{\rho }\left[\frac{a_{\text{2}}}{c_{\text{2}}}ln\left(1\text{+}T\right)+\frac{f_{\text{2}}-f_{R}}{c_{\text{2}}}\frac{l}{\rho +\delta }\right] \end{array}$$ (30)

Finding 3: The higher the development level of screening technology, the greater the efforts of government and social forces to prevent schistosomiasis in African children.

### 3.3 Case analysis

In order to describe the changes in social utility of government and social force in the process of preventing schistosomiasis in African children in more detail, this paper adopts the method of case analysis. This article takes the review of the schistosomiasis by African leaders at the African Union Summit as an example for case analysis. Case information content mainly comes from Global Schistosomiasis Alliance’s official website (https://www.eliminateschisto.org). The following assumptions are made for relevant parameters:

The screening process may be perceived as an infringement on individual privacy or freedom, especially in the absence of sufficient communication and community involvement. In some socio-cultural contexts, being screened or marked as a disease carrier could lead to social stigma or alienation, hence generating discontent and resistance. Furthermore, the allocation of screening resources might be uneven, with some groups feeling they have not received enough attention or support, exacerbating feelings of dissatisfaction [21]. This is particularly relevant in the context of neglected tropical diseases (NTDs), such as schistosomiasis, where sustained political commitment and domestic funding are essential for effective disease control programs. The African Leaders Malaria Alliance (ALMA), a coalition of African heads of state, has been instrumental in driving progress towards eliminating NTDs by fostering cross-border collaboration and maintaining political and financial momentum. Therefore, the “degree of discontent triggered by screening” tends to be high.

The dissemination of health knowledge directly enhances individuals’ awareness and capacity for disease prevention, making its immediate benefits easily recognized and commended by community members. Moreover, the spread of health knowledge can also encourage individuals and families to take proactive preventive measures, reducing disease incidence, with more significant and direct positive effects [20]. Community engagement and behavioral change are critical components of the global effort to control and eliminate NTDs. Hence, the “reputation gained in promoting health knowledge dissemination” is higher than that generated by “the number of public health facility units.”

In contrast, while public health facilities are crucial for improving overall health standards and addressing public health crises, their construction and maintenance often require long-term investments, and their effects might not be as immediately apparent as those of health knowledge dissemination. Additionally, the construction and benefits of public health facilities are often seen as the government’s responsibility and duty, leading to a slower accumulation of positive community reputation. Meanwhile, deficiencies or poor management of these facilities can easily become a focus of public dissatisfaction. This is evident in the fight against NTDs, where investments in water, sanitation, and hygiene (WASH) infrastructure are essential for long-term success. The ALMA initiative emphasizes the importance of such investments, alongside medical interventions, to achieve the goal of an Africa free from malaria and other NTDs by 2030.

Integrating these insights, it is clear that while screening and public health infrastructure are vital components of disease control, the role of health knowledge dissemination and community involvement cannot be overstated. The success of initiatives like ALMA in combating NTDs underscores the need for a holistic approach that combines medical, infrastructural, and educational strategies to achieve sustainable health outcomes. For convenience, this paper hypothesizes that the level *f*_*R*_ of discontent caused by screening is 3. The reputation *f*_*P*_ gained for promoting health literacy is 1.5. The reputation *f*_*B*_ generated by the number of units of public health facilities operating is 1.2.

Reputation decay is a quantifiable index that reflects the diminution in the public’s perception of a project or intervention over time. If the reputation decay value is low, it indicates that the positive reputation gained from the intervention or government efforts can be maintained in the public eye for a relatively long period without rapid decline. Such scenarios are likely to occur in projects with evident effects or high community recognition. For instance, the ALMA, a coalition of African heads of state, has demonstrated sustained positive reputation by maintaining political and financial momentum in the fight against NTDs like schistosomiasis. Discount rates are commonly used to calculate the present value of future benefits or outcomes. Setting a higher discount rate implies a relatively optimistic attitude towards future outcomes, considering that future benefits still hold considerable present value. This is commonly seen in interventions with significant and more certain long-term impacts. For example, the long-term investments in water, sanitation, and hygiene infrastructure, as emphasized by ALMA, are critical for the sustained control and elimination of NTDs, reflecting a higher discount rate due to their significant future benefits. Meanwhile, referring to other studies regarding the setting of this parameter is advised [27,50], this paper hypothesizes that decay *δ* of reputation is 0.1. The discount rate *ρ* that occurs over time is 0.9.

At the same time, the following assumptions are made about insignificant parameters in this paper. Cost *c*_*B*_ per unit of public health facility operation is 2. Prevalence *β* of schistosomiasis is 3. The positive effects *l* of reputation is 1. The level *T* of development of screening technology is 4. The reputation *f*_1_, *f*_2_ gained by government or social force for preventing schistosomiasis in African children at a unit level is 1.5.

This paper conducts a sensitivity analysis on the unit intervention costs associated with schistosomiasis prevention. Given that the resource consumption patterns and financial thresholds vary across the three measures—health education, public health infrastructure construction, and regular screening—even minor variations in unit costs within the numerical analysis can precipitate significant changes in the optimal control portfolio under equilibrium conditions. Through this analysis, the study quantitatively demonstrates how governments or societal actors should dynamically pivot between expensive yet long-lasting infrastructure projects and lower-cost but high-frequency health education or screening initiatives under scenarios of varying budget constraints or cost volatility (e.g., fluctuations in vaccine prices or rising equipment maintenance costs), thereby revealing the most cost-effective intervention pathways.

On the other hand, from the perspective of academic contribution and model robustness, examining the sensitivity of the social benefit equilibrium solution to unit costs significantly enhances the persuasiveness and practical utility of the paper. It demonstrates that the research conclusions are not predicated on a single, idealized cost assumption but are resilient to complex economic fluctuations in the real world. By comparing the specific impacts of cost changes on the magnitude of prevalence reduction and net social benefits, the article can precisely identify the “leverage points” within the model that are most sensitive to cost. This not only enriches the application dimensions of differential games in the public health domain but also provides flexible, data-driven theoretical support for the allocation of international aid funds and government fiscal planning aimed at controlling schistosomiasis among children in Africa, rendering the answer to “how to prevent more effectively” more actionable and practically relevant.

Regarding the specific implementation of the numerical analysis methods, this sensitivity analysis is typically performed by assigning unit cost parameters across the system’s steady state or ergodic time paths. This approach intuitively illustrates, through simulation plots, how variations in cost coefficients dynamically distort the phase diagrams of optimal strategies, thereby translating abstract game equilibria into visualized decision-making evidence and ensuring that the numerical results accurately reflect the non-linear impacts of cost structures on the social benefit system for schistosomiasis control.

When the cost *c*_1_, *c*_2_ to government or social force of preventing schistosomiasis in African children at a unit level is 2, this article can calculate the social benefits of government:


$$\overset{*}{\underset{P1}{V}}=1+0.64{\left(0.87a_{\text{1}}+2.61\right)}^{\text{2}}$$ (31)

$$\overset{*}{\underset{B1}{V}}=1+2.222{\left(0.25a_{\text{1}}+0.68\right)}^{\text{2}}$$ (32)

$$\overset{*}{\underset{R1}{V}}=1+1.111{\left(0.8a_{\text{1}}-0.75\right)}^{\text{2}}$$ (33)

**Fig 2:**
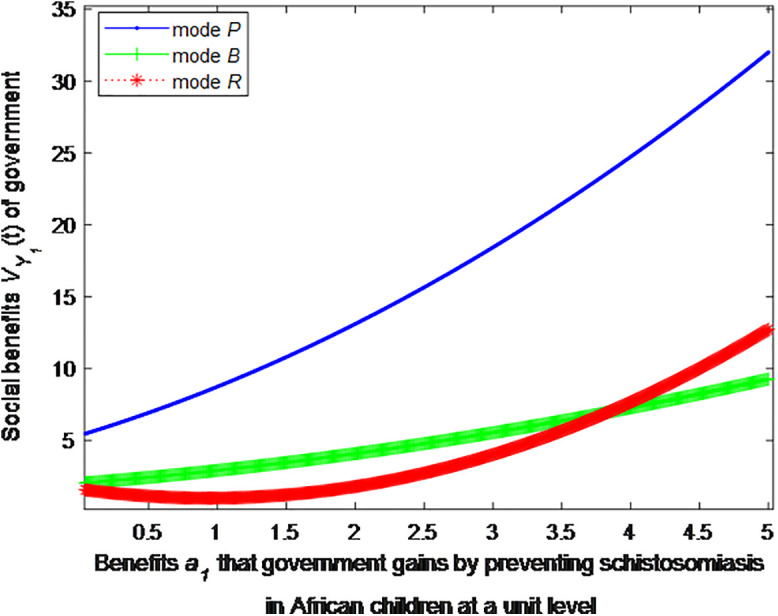
Impact of benefits of government on social welfare.

The following graph (named Fig 2) can also be produced:

When the cost *c*_1_, *c*_2_ to government or social force of preventing schistosomiasis in African children at a unit level is 5, this article can calculate the social benefits of government:


$$\overset{*}{\underset{P1}{V}}=1+1.6{\left(0.35a_{\text{1}}+1.04\right)}^{\text{2}}$$ (34)

$$\overset{*}{\underset{B1}{V}}=1+3.89{\left(0.143a_{\text{1}}+0.39\right)}^{\text{2}}$$ (35)

$$\overset{*}{\underset{R1}{V}}=1+2.78{\left(0.322a_{\text{1}}-0.3\right)}^{\text{2}}$$ (36)

**Fig 3:**
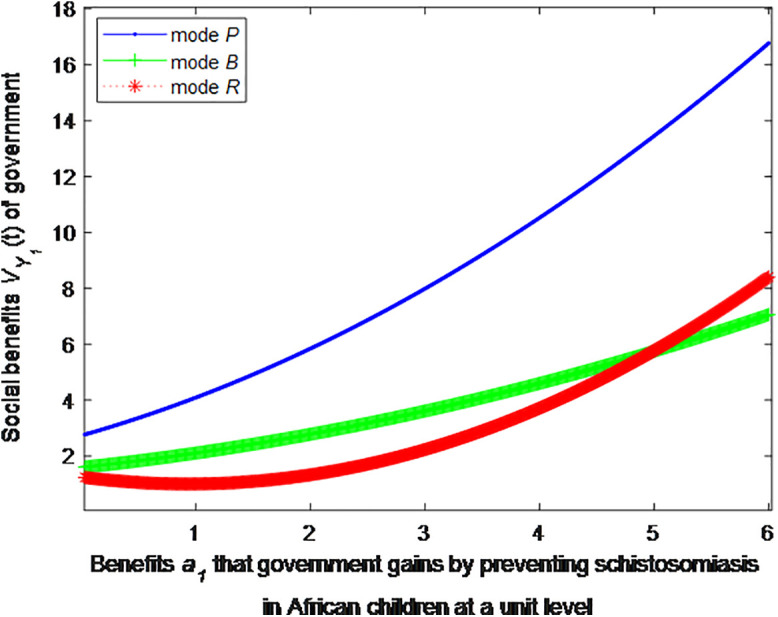
Impact of benefits of government on social welfare.

The following graph (named Fig 3) can also be produced:

Finding 4: When the benefits of schistosomiasis control per unit of population are small, the health education mode can provide the maximum social benefit, followed by the public health facility mode, and the regular screening mode provides the minimum social benefit. As the benefits of schistosomiasis control per unit of population increase, the social benefit of regular screening mode first decreases and then increases, and eventually exceeds the social benefit of the public health facility mode.

When the cost *c*_1_, *c*_2_ to government or social force of preventing schistosomiasis in African children at a unit level is 2, this article can calculate the social benefits of social force:


$$\overset{*}{\underset{P2}{V}}=1+1.111{\left(0.5a_{\text{2}}+1.5\right)}^{\text{2}}$$ (37)

$$\overset{*}{\underset{B2}{V}}=1+1.111{\left(0.5a_{\text{2}}+0.75\right)}^{\text{2}}$$ (38)

$$\overset{*}{\underset{R2}{V}}=1+1.111{\left(0.8a_{\text{2}}-0.75\right)}^{\text{2}}$$ (39)

**Fig 4:**
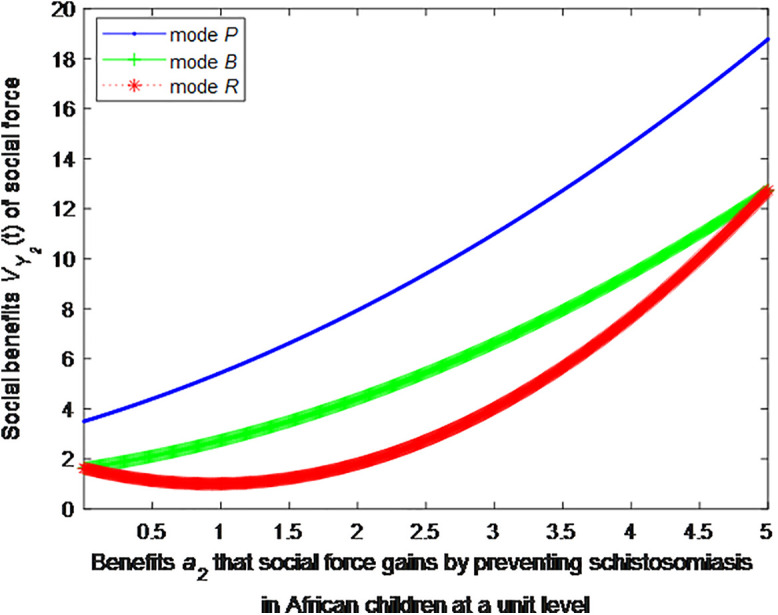
Impact of benefits of social force on social welfare.

**Fig 5:**
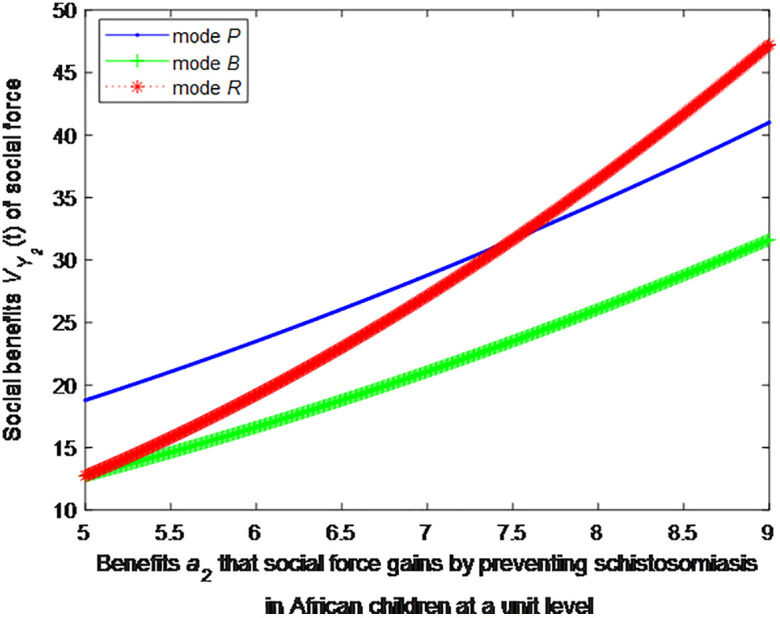
Impact of benefits of social force on social welfare.

The following graphs (named Fig 4 and Fig 5) can also be produced:

When the cost *c*_1_, *c*_2_ to government or social force of preventing schistosomiasis in African children at a unit level is 5, this article can calculate the social benefits of social force:


$$\overset{*}{\underset{P2}{V}}=1+2.78{\left(0.2a_{\text{2}}+0.6\right)}^{\text{2}}$$ (40)

$$\overset{*}{\underset{B2}{V}}=1+2.78{\left(0.2a_{\text{2}}+0.3\right)}^{\text{2}}$$ (41)

$$\overset{*}{\underset{R2}{V}}=1+2.78{\left(0.322a_{\text{2}}-0.3\right)}^{\text{2}}$$ (42)

**Fig 6:**
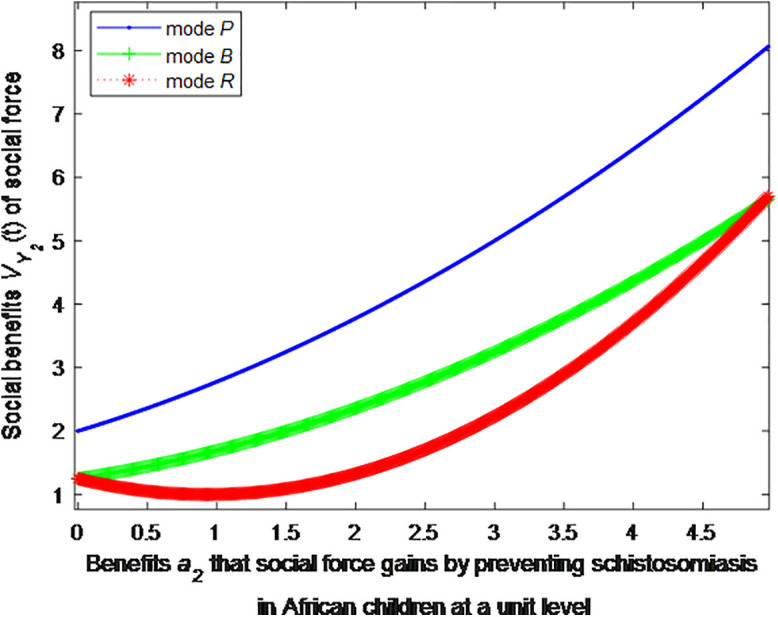
Impact of benefits of social force on social welfare.

**Fig 7:**
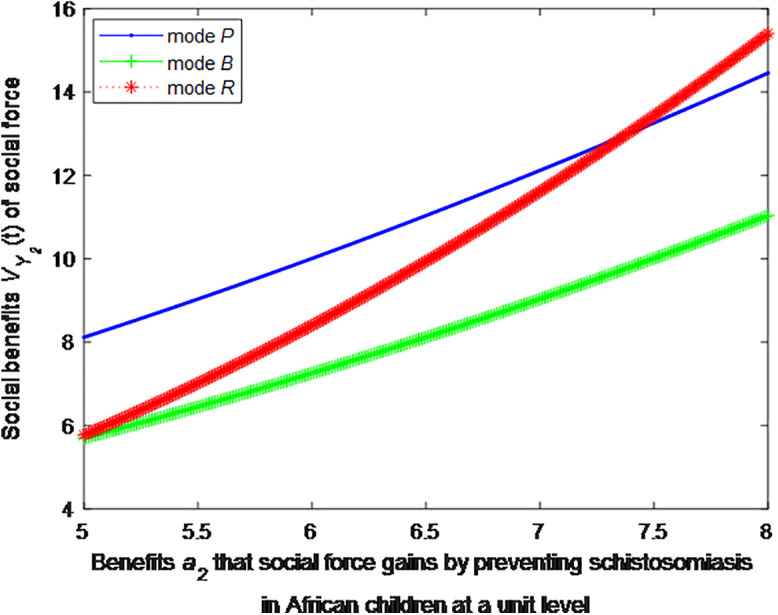
Impact of benefits of social force on social welfare.

The following graphs (named Fig 6 and Fig 7) can also be produced:

Finding 5: When the benefit of social forces in preventing schistosomiasis per unit is small, the health education mode can provide the maximum social benefit, followed by the public health facility mode, and the regular screening mode provides the minimum social benefit. As the benefit of social forces in preventing schistosomiasis per unit increases, the social benefit of regular screening mode first decreases and then increases. When the benefit of social forces in preventing schistosomiasis per unit increases to a certain extent, the social benefit of regular screening is the maximum.

## 4. Discussion

Finding 1 indicates that the prevalence of schistosomiasis is positively correlated with the government’s efforts in promoting health education, meaning that the higher the disease prevalence, the more inclined the government is to increase the promotion of health education. In addition, the reputation gained from popularizing health knowledge has a significant positive incentive effect on the prevention efforts of the government and social forces. The higher the reputation, the greater the investment of both in preventing schistosomiasis in African children. This indicates that reputation mechanisms play a key role in promoting the implementation of prevention strategies, while the popularization of health education has been proven to be an effective means of addressing high prevalence diseases. Finding 1 is mainly caused by the following reasons. Schistosomiasis is a disease caused by parasites, mainly transmitted through contact with contaminated water bodies. If the prevalence of schistosomiasis is high in an area, it means that a large number of people in the area are affected by the disease. This places a heavy burden on public health and socio-economic development. Therefore, in this case, the government will pay more attention to the investment and popularization of health education, in order to reduce the incidence of schistosomiasis and control the spread of the epidemic. The main purpose of investing government resources in health education is to improve the public’s understanding of schistosomiasis and educate people on how to prevent such diseases [2]. This may include the wide dissemination of information on the symptoms, transmission routes and prevention methods of schistosomiasis, so that people can take independent measures to avoid contact with contaminated water sources and reduce the risk of infection. In summary, the higher the prevalence of schistosomiasis is, the more incentive the government has to invest resources in health education, so as to improve public health and reduce the socio-economic burden by reducing the incidence and transmission of the disease by improving people’s health awareness. Childhood schistosomiasis in Africa is a serious public health problem that requires a large amount of resources and efforts to be properly addressed. Therefore, if governments and social forces have achieved a high reputation in the popularization of health knowledge, they are likely to have higher social trust and greater motivation to invest more resources and efforts in preventing and resisting the disease.

Based on Finding 1, policymakers, non-governmental organizations, and public health agencies should take the following actions. Policymakers should prioritize increased investment in health education, particularly in areas with high schistosomiasis prevalence, by formulating and implementing comprehensive health education programs to enhance public awareness and knowledge of disease prevention. Additionally, monitoring mechanisms should be established to dynamically adjust resource allocation based on the prevalence of the disease [51]. Non-governmental organizations should leverage their reputation and influence within communities to actively collaborate with the government in promoting the dissemination of health education. Through community mobilization and volunteer training, they should strengthen grassroots capacity for disease prevention. Public health agencies, on the other hand, need to integrate resources to enhance comprehensive interventions, including health education, public health infrastructure development, and regular screenings. They should also periodically evaluate the effectiveness of these interventions to ensure the efficient utilization of resources. The three parties should establish a collaborative mechanism to jointly reduce the incidence of schistosomiasis among African children and improve overall public health levels.

Finding 2 indicates that the higher the unit operating cost of public health facilities, the lower the government’s willingness to construct such facilities. This indicates that high operating costs will become an important constraint for government investment in public health facilities, which may lead to the government’s tendency to choose other more cost-effective prevention strategies, such as promoting health education or screening technologies. This discovery emphasizes the need to comprehensively consider the balance between cost and effectiveness when formulating strategies for preventing schistosomiasis. The reason of Finding 2 may be that the government needs to invest certain resources, including financial and human resources, in the construction and operation of public health facilities. When the operation cost of a single unit of public health facilities increases, it means that more resources are needed to support the operation of the facilities. In the case of limited resources, the government may need to make a trade-off between various areas and make a choice on resource allocation. The government may choose to reduce the construction of new public health facilities, invest more resources in the maintenance and operation of existing facilities, or find ways to reduce the operation cost, such as improving management methods, introducing new technologies, etc. On the other hand, if the operation cost of public health facilities is too high, it may first warn the government that the methods for schistosomiasis prevention in African children need to be re-evaluated and improved, and find more economical and effective solutions (Such as biological control programs) [52]. In summary, this sentence illustrates the negative correlation between the operation cost of public health facilities and the government’s efforts in their construction. That is, because of limited resources, higher operating costs for a single unit of public health facilities may lead to a relatively lower government effort to build new facilities.

Based on Finding 2, policymakers, non-governmental organizations, and public health agencies should take the following actions. Policymakers should optimize the operational costs of public health facilities by reducing unit costs through technological innovation, public-private partnerships, or resource sharing, thereby incentivizing the government to construct more public health facilities. Additionally, long-term planning should be formulated to ensure that facility construction aligns with disease prevention needs [53]. Non-governmental organizations can assist in lowering facility operational costs by providing technical support or financial aid, while also participating in the management and maintenance of these facilities to enhance resource utilization efficiency. Public health agencies, on the other hand, should strengthen cost-benefit analyses, prioritize the construction of cost-effective facilities, and promote multifunctional designs to meet other public health needs alongside schistosomiasis prevention. The three parties should collaborate closely to ensure the efficient construction and sustainable operation of public health facilities through cost optimization and resource integration, thereby more effectively preventing schistosomiasis among African children.

Finding 3 indicates that the higher the level of development of screening technology, the greater the investment of government and social forces in preventing schistosomiasis in African children. This indicates that advanced technology can improve the efficiency and accuracy of screening, thereby enhancing the effectiveness of preventive measures and motivating all parties to increase resource investment. This discovery emphasizes the important role of technological progress in disease prevention and indicates that promoting innovation and development of screening technology is one of the key strategies for effective prevention and control of schistosomiasis. Finding 3 is mainly caused by the following reasons. Screening technology is extremely important in the prevention and control of diseases. High-level screening technology can detect child patients with schistosomiasis earlier and more accurately, and provide timely treatment to prevent the deterioration of the disease or the spread of the disease, reducing the health burden of society. When the screening technology is continuously improved and developed, it will also encourage government and social forces to make greater efforts for the health of people [25]. On the one hand, the government may see the effect of high-level screening technology on the prevention and control of diseases, so as to increase investment, update and upgrade public health facilities, improve screening procedures, improve screening coverage, reduce omissions, and provide timely treatment for screened cases [27]. On the other hand, with the development of screening technology, more social forces may be attracted to participate in the prevention of schistosomiasis in African children. This may include private social forces, non-governmental organizations, volunteers, etc. They may work with the government to prevent and control schistosomiasis by continuously improving technology, providing funds, human resources, or through publicity and education. In general, this sentence emphasizes the positive correlation between the development level of screening technology and the efforts of the whole society in disease prevention, and takes schistosomiasis in African children as an example.

Based on Finding 3, policymakers, non-governmental organizations, and public health agencies should take the following actions. Policymakers should increase investment in the research, development, and promotion of screening technologies, support the application of innovative technologies, and drive the widespread adoption of efficient and cost-effective screening technologies in areas with high schistosomiasis prevalence through policy incentives and financial support. Non-governmental organizations should actively participate in the promotion and training of screening technologies, collaborating with the government to provide technical support and capacity building for grassroots healthcare workers, ensuring the effective implementation of screening technologies [54]. Public health agencies, on the other hand, need to integrate resources to establish advanced screening technology-based monitoring and response systems, conduct large-scale screening activities regularly, and utilize data analysis to optimize disease prevention strategies. The three parties should collaborate synergistically to enhance screening efficiency through technological upgrades and resource integration, thereby more effectively preventing and controlling the transmission of schistosomiasis among African children.

Finding 4 mainly elucidates the changes in social benefits of the government’s three prevention strategies (health education, public health facilities, and regular screening) under different conditions of schistosomiasis control benefits per unit population. Firstly, when the government unit revenue from schistosomiasis prevention is low, the universal health education mode brings the largest social benefits to the government. This is because health education usually costs little but can help the public understand the knowledge of disease prevention and control, thereby reducing the risk of infection [19]. The public health facility mode and regular screening mode require relatively high inputs, and their social benefits rank second and third respectively. Secondly, as the government unit revenue from schistosomiasis prevention increases, the social benefits from regular screening mode experience a process of first decreasing and then increasing. At the early stage of the increase in revenue, the social benefits may decrease due to the increase in inputs required for screening. However, as the screening technology is optimized and the coverage is expanded, this mode can more effectively detect and treat cases, thereby increasing the social benefits [24]. Finally, the social benefits from regular screening mode may exceed those from the public health facility mode. In short, Finding 4 analyzes the social benefits of different modes for governments at different levels of schistosomiasis prevention revenue. When the unit revenue is low, the universal health education mode is more advantageous; while with the increase of revenue, the regular screening mode gradually shows higher social benefits.

Based on Finding 4, policymakers, non-governmental organizations, and public health agencies should take the following actions. In regions with lower per capita benefits in schistosomiasis control, policymakers should prioritize the promotion of health education models, enhancing public awareness through the dissemination of disease prevention knowledge, while gradually constructing public health facilities as a supplementary measure. Non-governmental organizations should concentrate resources to support health education programs, strengthening grassroots educational capacity through community mobilization and training. Public health agencies, on the other hand, need to design low-cost, high-efficiency health education initiatives to ensure broad population coverage. In regions with higher per capita benefits, policymakers should increase investment in regular screening models, promote the application of advanced screening technologies, and optimize screening processes to improve efficiency. Non-governmental organizations can assist in the promotion and training of screening technologies to ensure their effective implementation. Public health agencies need to integrate resources to establish efficient screening and monitoring systems, dynamically adjusting strategies to maximize social benefits [55]. The three parties should flexibly select and optimize intervention models based on regional differences to ensure the efficient utilization of resources, thereby more effectively preventing schistosomiasis among African children.

Finding 5 describes the social benefits of the three modes (universal health education mode, public health facility mode and regular screening mode) for social forces at different levels of schistosomiasis prevention benefits. Firstly, when the unit benefits of schistosomiasis prevention of social forces are low, the social benefits of universal health education mode are the largest. This is because, through publicity and education, this mode can let the public understand the harm of schistosomiasis, prevention and control methods, and improve the public’s awareness of self-protection, which makes the input of social forces achieve higher benefits [13]. The social benefits of the public health facility mode and the regular screening mode are higher, ranking second and third. Then, with the gradual increase of the unit benefits of schistosomiasis prevention of social forces, the social benefits of the regular screening mode will experience a process of first decreasing and then increasing. At the beginning, due to the increase of input costs, the social benefits may decrease. However, with the improvement of screening technology and scope, the regular screening mode can more effectively identify local patients and imported patients, thereby improving the social benefits [56]. Finally, when the unit-level benefits of schistosomiasis prevention by social forces increase to a certain extent, the social benefits obtained by social forces under the regular screening mode will reach the maximum. This means that at this stage, the role of social forces in schistosomiasis prevention and control through the regular screening mode can be better played, thus creating greater value for the entire society. In summary, Finding 5 analyzes the different modes that social forces should choose under different levels of benefits of schistosomiasis prevention to obtain the maximum social benefits. When the unit benefits are low, the universal health education mode is more advantageous; while with the increase of benefits, the social benefits of the regular screening mode gradually become prominent. Once the benefits reach a certain level, the regular screening mode will bring the maximum social benefits.

Based on Research Finding 5, policymakers, non-governmental organizations, and public health agencies should take the following actions. When the unit benefit of social forces in schistosomiasis prevention is low, policymakers should prioritize the promotion of health education models, enhancing public awareness through the dissemination of disease prevention knowledge, while gradually constructing public health facilities as a supplementary measure. Non-governmental organizations should concentrate resources to support health education programs, strengthening grassroots educational capacity through community mobilization and training. Public health agencies, on the other hand, need to design low-cost, high-efficiency health education initiatives to ensure broad population coverage. When the unit benefit of social forces significantly improves, policymakers should increase investment in regular screening models, promote the application of advanced screening technologies, and optimize screening processes to improve efficiency. Non-governmental organizations can assist in the promotion and training of screening technologies to ensure their effective implementation. Public health agencies need to integrate resources to establish efficient screening and monitoring systems, dynamically adjusting strategies to maximize social benefits [55]. The three parties should flexibly select and optimize intervention models based on changes in the benefit of social forces to ensure the efficient utilization of resources, thereby more effectively preventing schistosomiasis among African children.

In this paper, it is demonstrated that the social benefits of periodic screening models are maximized when the effectiveness of schistosomiasis prevention at the unit level reaches a certain threshold. However, the ethical issues involved in the implementation of periodic screening models, such as privacy, informed consent, and the risk of stigmatization, must be given full attention. To make screening culturally acceptable and ethically justified, successful experiences and lessons from HIV and tuberculosis (TB) screening in Africa can be referenced. For instance, in HIV screening, many African countries have gradually alleviated fears and stigmatization through community involvement and culturally sensitive educational approaches [57]. By integrating screening with local cultural practices, such as utilizing religious leaders or community elders as advocates, trust and acceptance of screening within the community can be enhanced. Additionally, ensuring privacy protection and informed consent during the screening process, such as through anonymous testing and providing clear screening information, is also crucial for increasing participation rates [58].

In terms of ethics, this paper should emphasize that screening must be conducted on the basis of respecting individual rights. Taking TB screening as an example, many African countries have successfully reduced ethical controversies in the screening process by establishing strict privacy protection mechanisms and providing adequate informed consent procedures. For instance, in TB screening, ensuring the confidentiality of test results and providing follow-up treatment and support services help alleviate the psychological burden and risk of stigmatization among participants [59]. Therefore, this paper should recommend adopting similar measures in schistosomiasis screening, such as eliminating misunderstandings about the disease through community health education, establishing transparent screening processes, and providing follow-up treatment and counseling services to ensure that screening meets ethical requirements and is widely accepted by the community. By drawing on the experiences of HIV and TB screening, this paper can provide a more culturally adaptive and ethically reasonable framework for the implementation of schistosomiasis screening.

The differential game model employed in this study, while capable of analyzing the dynamic interactions and long-term effects of different strategies through mathematical modeling, has certain limitations. First, differential game models often rely on simplified assumptions and idealized conditions, which may not fully capture the complexity and uncertainty of the real world. For instance, the model might assume uniform responses across individuals to health education, facility utilization, and screening, whereas in reality, significant variations in responses can arise due to differences in regions, cultural backgrounds, and economic conditions. Additionally, the model may struggle to accurately quantify certain factors, such as changes in social behavior, the intensity of policy implementation, and environmental influences, all of which can impact the actual effectiveness of prevention strategies. Second, the results of the differential game model are highly dependent on parameter settings and initial condition assumptions. If parameter estimates are inaccurate or initial conditions do not align with real-world scenarios, the model’s predictions may exhibit bias.

In this paper, although we assume that health education, the construction of public health facilities, and periodic screening can be directly implemented, in practice, financial constraints and community hesitancy are two challenges that cannot be overlooked. First, the limited health budgets in many regions of Africa make it difficult to cover large-scale health education programs, facility construction, and screening activities. Particularly in resource-scarce areas, prioritizing investments in infrastructure development may squeeze funding for other critical health services, thereby increasing the vulnerability of the overall health system [60]. Therefore, future research should explicitly consider how to optimize resource allocation, such as through public-private partnerships, international aid, or community fundraising, to alleviate financial pressures.

Second, community hesitancy and potential resistance are also significant obstacles to the implementation of preventive measures. Due to misunderstandings about the disease, cultural taboos, or fears about the screening process, some communities may be reluctant to participate in health education and screening activities [61]. Future research should explore how to gradually eliminate these barriers through community engagement, culturally sensitive educational approaches, and trust-building mechanisms. For example, in the future, the guidance of local or religious leaders could enhance community acceptance of preventive measures, while ensuring that health education content aligns with local cultural contexts to reduce misunderstandings and resistance.

## 5. Conclusion

The problem of schistosomiasis in African children is outstanding. In order to effectively prevent schistosomiasis in African children, this paper established a differential game model of three modes: universal health education, construction of public health facilities and regular screening. At the same time, this paper calculated the applicable range of various differential game modes. Finally, the research conclusions are drawn: when the benefits obtained by the government and social forces in preventing schistosomiasis at unit level are small, the universal health education mode can make the government and social forces obtain the maximum social benefits, the establishment of public health facilities mode comes next, and the regular screening mode makes the government and social forces obtain the minimum social benefits. With the increase of the benefits obtained by the government and social forces in preventing schistosomiasis at unit level, the social benefits obtained by the government and social forces in the regular screening mode first decrease and then increase. When the benefits obtained by the government and social forces in preventing schistosomiasis at unit level increase to a certain extent, the social benefits obtained by the social forces under the regular screening are the maximum.

The research in this paper can also be extended. For example, the following assumptions are made in this paper: the cost of governmental health education universalization is affected by the initial universalization level; it takes costs to operate public health facilities; and advances in screening technology will improve the efficiency of screening. In future research, these assumptions can be removed for further study. Meanwhile, some blanks in this study can also be filled in in future research. First, the specific standards to be adopted by governmental and social forces in the process of schistosomiasis prevention in African children under different conditions should be determined. Second, the results of schistosomiasis prevention in African children will be translated into feasible policy recommendations for reference by governmental and social forces. Third, in the process of schistosomiasis prevention in African children, governmental and social forces should determine the order of action of relevant research, rather than taking action simultaneously.

## Supporting information

S1 File (DOCX)

S2 File (DOCX)

S3 File (DOCX)
