# Supplementary material for: Popularizing health education, building public health facilities or regular screening? How to prevent schistosomiasis more effectively in African children
Source: PLoS One. 2026 Apr 20;21(4):e0347325. doi: 10.1371/journal.pone.0347325 (PMC13095015; doi:10.1371/journal.pone.0347325)
Supplement: S1 File — (DOCX) [file pone.0347325.s001.docx]

**Proof of (19) - (22)**

Take the derivatives of *F_P_*_1_ with respect to (13), and take the derivatives of *F_P_*_2_ with respect to (14), and set them equal to zero, we can get:

$F_{P1}^{*}\left(t\right)=\frac{a_{1}}{c_{1}}\ln\left(\text{e+}\beta\right)+\frac{f_{1}+f_{P}}{c_{1}}\frac{\partial V_{P1}}{\partial x_{P1}}\ln\left(\text{e+}\beta\right)$ (43)

$F_{P2}^{*}\left(t\right)=\frac{a_{2}}{c_{2}}+\frac{f_{2}+f_{P}}{c_{2}}\frac{\partial V_{P2}}{\partial x_{P2}}$ (44)

Substituting (43) into (13) and substituting (44) into (14), we can get:

$\begin{matrix} \rho V_{P1}=a_{1}\left[\frac{a_{1}}{c_{1}}\ln\left(\text{e+}\beta\right)+\frac{f_{1}+f_{P}}{c_{1}}\frac{\partial V_{P1}}{\partial x_{P1}}\ln\left(\text{e+}\beta\right)\right]-\frac{c_{1}}{2\ln\left(\text{e+}\beta\right)}\left[\frac{a_{1}}{c_{1}}\ln\left(\text{e+}\beta\right)+\frac{f_{1}+f_{P}}{c_{1}}\frac{\partial V_{P1}}{\partial x_{P1}}\ln\left(\text{e+}\beta\right)\right]^{2} \\ +lx_{P1}\left(t\right)+\frac{\partial V_{P1}}{\partial x_{P1}}\left(f_{1}+f_{P}\right)\left[\frac{a_{1}}{c_{1}}\ln\left(\text{e+}\beta\right)+\frac{f_{1}+f_{P}}{c_{1}}\frac{\partial V_{P1}}{\partial x_{P1}}\ln\left(\text{e+}\beta\right)\right]-\frac{\partial V_{P1}}{\partial x_{P1}}\delta x_{P1}\left(t\right) \end{matrix}$(45)

$\begin{matrix} \rho V_{P2}=a_{2}\left(\frac{a_{2}}{c_{2}}+\frac{f_{2}+f_{P}}{c_{2}}\frac{\partial V_{P2}}{\partial x_{P2}}\right)-\frac{c_{2}}{2}\left(\frac{a_{2}}{c_{2}}+\frac{f_{2}+f_{P}}{c_{2}}\frac{\partial V_{P2}}{\partial x_{P2}}\right)^{2}+lx_{P2}\left(t\right) \\ +\frac{\partial V_{P2}}{\partial x_{P2}}\left[\left(f_{2}+f_{P}\right)\left(\frac{a_{2}}{c_{2}}+\frac{f_{2}+f_{P}}{c_{2}}\frac{\partial V_{P2}}{\partial x_{P2}}\right)-\delta x_{P2}\left(t\right)\right] \end{matrix}$ (46)

Let $V_{P1}^{*}=k_{1}x_{P1}+k_{2}$, $V_{P2}^{\text{*}}=k_{3}x_{P2}+k_{4}$, wherein, *k*_1_, *k*_2_, *k*_3_ and *k*_4_ are all constants. The parameters of the optimal social welfare function can be obtained by calculation as follows:

$\left\{\begin{matrix} k_{1}=\frac{l}{\rho+\delta} \\ k_{2}=\frac{1}{\rho}a_{1}\left[\frac{a_{1}}{c_{1}}\ln\left(\text{e+}\beta\right)+\frac{f_{1}+f_{P}}{c_{1}}\frac{l}{\rho+\delta}\ln\left(\text{e+}\beta\right)\right]-\left[\frac{a_{1}}{c_{1}}\ln\left(\text{e+}\beta\right)+\frac{f_{1}+f_{P}}{c_{1}}\frac{l}{\rho+\delta}\ln\left(\text{e+}\beta\right)\right]^{2} \\ \frac{c_{1}}{2\ln\left(\text{e+}\beta\right)}\frac{1}{\rho}+\frac{1}{\rho}\frac{l}{\rho+\delta}\left(f_{1}+f_{P}\right)\left[\frac{a_{1}}{c_{1}}\ln\left(\text{e+}\beta\right)+\frac{f_{1}+f_{P}}{c_{1}}\frac{l}{\rho+\delta}\ln\left(\text{e+}\beta\right)\right] \end{matrix}\right.$ (47)

$\left\{\begin{matrix} k_{3}=\frac{l}{\rho+\delta} \\ k_{4}=\frac{1}{\rho}a_{2}\left(\frac{a_{2}}{c_{2}}+\frac{f_{2}+f_{P}}{c_{2}}\frac{l}{\rho+\delta}\right)-\frac{c_{2}}{2}\frac{1}{\rho}\left(\frac{a_{2}}{c_{2}}+\frac{f_{2}+f_{P}}{c_{2}}\frac{l}{\rho+\delta}\right)^{2} \\ +\frac{l}{\rho+\delta}\frac{1}{\rho}\left(f_{2}+f_{P}\right)\left(\frac{a_{2}}{c_{2}}+\frac{f_{2}+f_{P}}{c_{2}}\frac{l}{\rho+\delta}\right) \end{matrix}\right.$ (48)

Therefore, it can be concluded that:

$\begin{matrix} V_{P1}^{*}=\frac{1}{\rho}a_{1}\left[\frac{a_{1}}{c_{1}}\ln\left(\text{e+}\beta\right)+\frac{f_{1}+f_{P}}{c_{1}}\frac{l}{\rho+\delta}\ln\left(\text{e+}\beta\right)\right]-\left[\frac{a_{1}}{c_{1}}\ln\left(\text{e+}\beta\right)+\frac{f_{1}+f_{P}}{c_{1}}\frac{l}{\rho+\delta}\ln\left(\text{e+}\beta\right)\right]^{2} \\ \frac{c_{1}}{2\ln\left(\text{e+}\beta\right)}\frac{1}{\rho}+\frac{1}{\rho}\frac{l}{\rho+\delta}\left(f_{1}+f_{P}\right)\left[\frac{a_{1}}{c_{1}}\ln\left(\text{e+}\beta\right)+\frac{f_{1}+f_{P}}{c_{1}}\frac{l}{\rho+\delta}\ln\left(\text{e+}\beta\right)\right]+\frac{l}{\rho+\delta}x_{P1} \end{matrix}$ (49)

$\begin{matrix} V_{P2}^{\text{*}}=\frac{l}{\rho+\delta}x_{P2}+\frac{1}{\rho}a_{2}\left(\frac{a_{2}}{c_{2}}+\frac{f_{2}+f_{P}}{c_{2}}\frac{l}{\rho+\delta}\right)-\frac{c_{2}}{2}\frac{1}{\rho}\left(\frac{a_{2}}{c_{2}}+\frac{f_{2}+f_{P}}{c_{2}}\frac{l}{\rho+\delta}\right)^{2} \\ +\frac{l}{\rho+\delta}\frac{1}{\rho}\left(f_{2}+f_{P}\right)\left(\frac{a_{2}}{c_{2}}+\frac{f_{2}+f_{P}}{c_{2}}\frac{l}{\rho+\delta}\right) \end{matrix}$ (50)

In this case,

$F_{P1}^{*}\left(t\right)=\frac{a_{1}}{c_{1}}\ln\left(\text{e+}\beta\right)+\frac{f_{1}+f_{P}}{c_{1}}\frac{l}{\rho+\delta}\ln\left(\text{e+}\beta\right)$ (51)

$F_{P2}^{*}\left(t\right)=\frac{a_{2}}{c_{2}}+\frac{f_{2}+f_{P}}{c_{2}}\frac{l}{\rho+\delta}$ (52)
