# Supplementary material for: Popularizing health education, building public health facilities or regular screening? How to prevent schistosomiasis more effectively in African children
Source: PLoS One. 2026 Apr 20;21(4):e0347325. doi: 10.1371/journal.pone.0347325 (PMC13095015; doi:10.1371/journal.pone.0347325)
Supplement: S2 File — (DOCX) [file pone.0347325.s002.docx]

**Proof of (23) - (26)**

Take the derivatives of *F_B_*_1_ with respect to (15), and take the derivatives of *F_B_*_2_ with respect to (16), and set them equal to zero, we can get:

$F_{B1}^{*}\left(t\right)=\frac{a_{1}}{c_{1}+c_{B}}+\frac{f_{1}+f_{B}}{c_{1}+c_{B}}\frac{\partial V_{B1}}{\partial x_{B1}}$ (53)

$F_{B2}^{*}\left(t\right)=\frac{a_{2}}{c_{2}}+\frac{f_{2}}{c_{2}}\frac{\partial V_{B2}}{\partial x_{B2}}$ (54)

Substituting (53) into (15) and substituting (54) into (16), we can get:

$\begin{matrix} \rho V_{B1}=a_{1}\left(\frac{a_{1}}{c_{1}+c_{B}}+\frac{f_{1}+f_{B}}{c_{1}+c_{B}}\frac{\partial V_{B1}}{\partial x_{B1}}\right)-\frac{\left(c_{1}+c_{B}\right)}{2}\left(\frac{a_{1}}{c_{1}+c_{B}}+\frac{f_{1}+f_{B}}{c_{1}+c_{B}}\frac{\partial V_{B1}}{\partial x_{B1}}\right)^{2} \\ +lx_{B1}\left(t\right)+\frac{\partial V_{B1}}{\partial x_{B1}}\left[\left(f_{1}+f_{B}\right)\left(\frac{a_{1}}{c_{1}+c_{B}}+\frac{f_{1}+f_{B}}{c_{1}+c_{B}}\frac{\partial V_{B1}}{\partial x_{B1}}\right)-\delta x_{B1}\left(t\right)\right] \end{matrix}$ (55)

$\rho V_{B2}=a_{2}\left(\frac{a_{2}}{c_{2}}+\frac{f_{2}}{c_{2}}\frac{\partial V_{B2}}{\partial x_{B2}}\right)-\frac{c_{2}}{2}\left(\frac{a_{2}}{c_{2}}+\frac{f_{2}}{c_{2}}\frac{\partial V_{B2}}{\partial x_{B2}}\right)^{2}+lx_{B2}\left(t\right)+\frac{\partial V_{B2}}{\partial x_{B2}}\left[f_{2}\left(\frac{a_{2}}{c_{2}}+\frac{f_{2}}{c_{2}}\frac{\partial V_{B2}}{\partial x_{B2}}\right)-\delta x_{B2}\left(t\right)\right]$ (56)

Let $V_{B1}^{*}=k_{5}x_{B1}+k_{6}$,$V_{B2}^{\text{*}}=k_{7}x_{B2}+k_{8}$, wherein, *k*_5_, *k*_6_, *k*_7_ and *k*_8_ are all constants. The parameters of the optimal social welfare function can be obtained by calculation as follows:

$\left\{\begin{matrix} k_{5}=\frac{l}{\rho+\delta} \\ k_{6}=\frac{1}{\rho}a_{1}\left(\frac{a_{1}}{c_{1}+c_{B}}+\frac{f_{1}+f_{B}}{c_{1}+c_{B}}\frac{l}{\rho+\delta}\right)-\frac{\left(c_{1}+c_{B}\right)}{2}\frac{1}{\rho}\left(\frac{a_{1}}{c_{1}+c_{B}}+\frac{f_{1}+f_{B}}{c_{1}+c_{B}}\frac{l}{\rho+\delta}\right)^{2} \\ +\frac{1}{\rho}\frac{l}{\rho+\delta}\left(f_{1}+f_{B}\right)\left(\frac{a_{1}}{c_{1}+c_{B}}+\frac{f_{1}+f_{B}}{c_{1}+c_{B}}\frac{l}{\rho+\delta}\right) \end{matrix}\right.$ (57)

$\left\{\begin{matrix} k_{7}=\frac{l}{\rho+\delta} \\ k_{8}=\frac{1}{\rho}a_{2}\left(\frac{a_{2}}{c_{2}}+\frac{f_{2}}{c_{2}}\frac{l}{\rho+\delta}\right)-\frac{c_{2}}{2}\frac{1}{\rho}\left(\frac{a_{2}}{c_{2}}+\frac{f_{2}}{c_{2}}\frac{l}{\rho+\delta}\right)^{2}+\frac{1}{\rho}\frac{l}{\rho+\delta}f_{2}\left(\frac{a_{2}}{c_{2}}+\frac{f_{2}}{c_{2}}\frac{l}{\rho+\delta}\right) \end{matrix}\right.$ (58)

Therefore, it can be concluded that:

$\begin{matrix} V_{B1}^{*}=\frac{l}{\rho+\delta}x_{B1}+\frac{1}{\rho}a_{1}\left(\frac{a_{1}}{c_{1}+c_{B}}+\frac{f_{1}+f_{B}}{c_{1}+c_{B}}\frac{l}{\rho+\delta}\right)-\frac{\left(c_{1}+c_{B}\right)}{2}\frac{1}{\rho}\left(\frac{a_{1}}{c_{1}+c_{B}}+\frac{f_{1}+f_{B}}{c_{1}+c_{B}}\frac{l}{\rho+\delta}\right)^{2} \\ +\frac{1}{\rho}\frac{l}{\rho+\delta}\left(f_{1}+f_{B}\right)\left(\frac{a_{1}}{c_{1}+c_{B}}+\frac{f_{1}+f_{B}}{c_{1}+c_{B}}\frac{l}{\rho+\delta}\right) \end{matrix}$ (59)

$V_{B2}^{\text{*}}=\frac{l}{\rho+\delta}x_{B2}+\frac{1}{\rho}a_{2}\left(\frac{a_{2}}{c_{2}}+\frac{f_{2}}{c_{2}}\frac{l}{\rho+\delta}\right)-\frac{c_{2}}{2}\frac{1}{\rho}\left(\frac{a_{2}}{c_{2}}+\frac{f_{2}}{c_{2}}\frac{l}{\rho+\delta}\right)^{2}+\frac{1}{\rho}\frac{\partial V_{B2}}{\partial x_{B2}}f_{2}\left(\frac{a_{2}}{c_{2}}+\frac{f_{2}}{c_{2}}\frac{l}{\rho+\delta}\right)$ (60)

In this case,

$F_{B1}^{*}\left(t\right)=\frac{a_{1}}{c_{1}+c_{B}}+\frac{f_{1}+f_{B}}{c_{1}+c_{B}}\frac{l}{\rho+\delta}$ (61)

$F_{B2}^{*}\left(t\right)=\frac{a_{2}}{c_{2}}+\frac{f_{2}}{c_{2}}\frac{l}{\rho+\delta}$ (62)
