# Supplementary material for: Popularizing health education, building public health facilities or regular screening? How to prevent schistosomiasis more effectively in African children
Source: PLoS One. 2026 Apr 20;21(4):e0347325. doi: 10.1371/journal.pone.0347325 (PMC13095015; doi:10.1371/journal.pone.0347325)
Supplement: S3 File — (DOCX) [file pone.0347325.s003.docx]

**Proof of (27) - (30)**

Take the derivatives of *F_R_*_1_ with respect to (17), and take the derivatives of *F_R_*_2_ with respect to (18), and set them equal to zero, we can get:

$F_{R1}^{*}\left(t\right)=\frac{a_{1}}{c_{1}}\ln\left(1\text{+}T\right)+\frac{f_{1}-f_{R}}{c_{1}}\frac{\partial V_{R1}}{\partial x_{R1}}$ (63)

$F_{R2}^{*}\left(t\right)=\frac{a_{2}}{c_{2}}\ln\left(1\text{+}T\right)+\frac{f_{2}-f_{R}}{c_{2}}\frac{\partial V_{R2}}{\partial x_{R2}}$ (64)

Substituting (63) into (17) and substituting (64) into (18), we can get:

$\begin{matrix} \rho V_{R1}=a_{1}\ln\left(1\text{+}T\right)\left[\frac{a_{1}}{c_{1}}\ln\left(1\text{+}T\right)+\frac{f_{1}-f_{R}}{c_{1}}\frac{\partial V_{R1}}{\partial x_{R1}}\right]-\frac{c_{1}}{2}\left[\frac{a_{1}}{c_{1}}\ln\left(1\text{+}T\right)+\frac{f_{1}-f_{R}}{c_{1}}\frac{\partial V_{R1}}{\partial x_{R1}}\right]^{2} \\ +lx_{R1}\left(t\right)+\frac{\partial V_{R1}}{\partial x_{R1}}\left(f_{1}-f_{R}\right)\left[\frac{a_{1}}{c_{1}}\ln\left(1\text{+}T\right)+\frac{f_{1}-f_{R}}{c_{1}}\frac{\partial V_{R1}}{\partial x_{R1}}\right]-\frac{\partial V_{R1}}{\partial x_{R1}}\delta x_{R1}\left(t\right) \end{matrix}$ (65)

$\begin{matrix} \rho V_{R2}=a_{2}\ln\left(1\text{+}T\right)\left[\frac{a_{2}}{c_{2}}\ln\left(1\text{+}T\right)+\frac{f_{2}-f_{R}}{c_{2}}\frac{\partial V_{R2}}{\partial x_{R2}}\right]-\frac{c_{2}}{2}\left[\frac{a_{2}}{c_{2}}\ln\left(1\text{+}T\right)+\frac{f_{2}-f_{R}}{c_{2}}\frac{\partial V_{R2}}{\partial x_{R2}}\right]^{2} \\ +lx_{R2}\left(t\right)+\frac{\partial V_{R2}}{\partial x_{R2}}\left(f_{2}-f_{R}\right)\left[\frac{a_{2}}{c_{2}}\ln\left(1\text{+}T\right)+\frac{f_{2}-f_{R}}{c_{2}}\frac{\partial V_{R2}}{\partial x_{R2}}\right]-\frac{\partial V_{R2}}{\partial x_{R2}}\delta x_{R2}\left(t\right) \end{matrix}$ (66)

Let $V_{R1}^{*}=k_{9}x_{R1}+k_{10}$,$V_{R2}^{\text{*}}=k_{11}x_{R2}+k_{12}$, wherein, *k*_9_, *k*_10_, *k*_11_ and *k*_12_ are all constants. The parameters of the optimal social welfare function can be obtained by calculation as follows:

$\left\{\begin{matrix} k_{9}=\frac{l}{\rho+\delta} \\ k_{10}=\frac{1}{\rho}a_{1}\ln\left(1\text{+}T\right)\left[\frac{a_{1}}{c_{1}}\ln\left(1\text{+}T\right)+\frac{f_{1}-f_{R}}{c_{1}}\frac{l}{\rho+\delta}\right]-\frac{c_{1}}{2}\frac{1}{\rho}\left[\frac{a_{1}}{c_{1}}\ln\left(1\text{+}T\right)+\frac{f_{1}-f_{R}}{c_{1}}\frac{l}{\rho+\delta}\right]^{2} \\ +\frac{l}{\rho+\delta}\frac{1}{\rho}\left(f_{1}-f_{R}\right)\left[\frac{a_{1}}{c_{1}}\ln\left(1\text{+}T\right)+\frac{f_{1}-f_{R}}{c_{1}}\frac{l}{\rho+\delta}\right] \end{matrix}\right.$ (67)

$\left\{\begin{matrix} k_{11}=\frac{l}{\rho+\delta} \\ k_{12}=\frac{1}{\rho}a_{2}\ln\left(1\text{+}T\right)\left[\frac{a_{2}}{c_{2}}\ln\left(1\text{+}T\right)+\frac{f_{2}-f_{R}}{c_{2}}\frac{l}{\rho+\delta}\right]-\frac{c_{2}}{2}\frac{1}{\rho}\left[\frac{a_{2}}{c_{2}}\ln\left(1\text{+}T\right)+\frac{f_{2}-f_{R}}{c_{2}}\frac{l}{\rho+\delta}\right]^{2} \\ +\frac{l}{\rho+\delta}\left(f_{2}-f_{R}\right)\frac{1}{\rho}\left[\frac{a_{2}}{c_{2}}\ln\left(1\text{+}T\right)+\frac{f_{2}-f_{R}}{c_{2}}\frac{l}{\rho+\delta}\right] \end{matrix}\right.$ (68)

Therefore, it can be concluded that:

$\begin{matrix} V_{R1}^{*}=\frac{l}{\rho+\delta}x_{R1}+\frac{1}{\rho}a_{1}\ln\left(1\text{+}T\right)\left[\frac{a_{1}}{c_{1}}\ln\left(1\text{+}T\right)+\frac{f_{1}-f_{R}}{c_{1}}\frac{l}{\rho+\delta}\right]-\frac{c_{1}}{2}\frac{1}{\rho}\left[\frac{a_{1}}{c_{1}}\ln\left(1\text{+}T\right)+\frac{f_{1}-f_{R}}{c_{1}}\frac{l}{\rho+\delta}\right]^{2} \\ +\frac{l}{\rho+\delta}\frac{1}{\rho}\left(f_{1}-f_{R}\right)\left[\frac{a_{1}}{c_{1}}\ln\left(1\text{+}T\right)+\frac{f_{1}-f_{R}}{c_{1}}\frac{l}{\rho+\delta}\right] \end{matrix}$(69)

$\begin{matrix} V_{R2}^{\text{*}}=\frac{l}{\rho+\delta}x_{R2}+\frac{1}{\rho}a_{2}\ln\left(1\text{+}T\right)\left[\frac{a_{2}}{c_{2}}\ln\left(1\text{+}T\right)+\frac{f_{2}-f_{R}}{c_{2}}\frac{l}{\rho+\delta}\right]-\frac{c_{2}}{2}\frac{1}{\rho}\left[\frac{a_{2}}{c_{2}}\ln\left(1\text{+}T\right)+\frac{f_{2}-f_{R}}{c_{2}}\frac{l}{\rho+\delta}\right]^{2} \\ +\frac{l}{\rho+\delta}\left(f_{2}-f_{R}\right)\frac{1}{\rho}\left[\frac{a_{2}}{c_{2}}\ln\left(1\text{+}T\right)+\frac{f_{2}-f_{R}}{c_{2}}\frac{l}{\rho+\delta}\right] \end{matrix}$(70)

In this case,

$F_{R1}^{*}\left(t\right)=\frac{a_{1}}{c_{1}}\ln\left(1\text{+}T\right)+\frac{f_{1}-f_{R}}{c_{1}}\frac{l}{\rho+\delta}$ (71)

$F_{R2}^{*}\left(t\right)=\frac{a_{2}}{c_{2}}\ln\left(1\text{+}T\right)+\frac{f_{2}-f_{R}}{c_{2}}\frac{l}{\rho+\delta}$ (72)
